# Author Correction: Phenotypes associated with genes encoding drug targets are predictive of clinical trial side effects

**DOI:** 10.1038/s41467-019-10237-6

**Published:** 2019-05-08

**Authors:** Phuong A. Nguyen, David A. Born, Aimee M. Deaton, Paul Nioi, Lucas D. Ward

**Affiliations:** 1 Amgen, Inc., 360 Binney St., Cambridge, MA 02142 USA; 2 Alnylam Pharmaceuticals, Inc., 300 Third St., Cambridge, MA 02142 USA

**Keywords:** Computational biology and bioinformatics, Drug safety, Genetics, Systems biology

Correction to: *Nature Communications* 10.1038/s41467-019-09407-3, published online 5 April 2019.

In the original version of this article, there were errors in the labelling of the colours in the key of Fig. 2, whereby the labeling of the third and fourth of the four colours was reversed. This has been corrected in both the PDF and HTML versions of the article.

