# Supplementary material for: Phenotypes associated with genes encoding drug targets are predictive of clinical trial side effects
Source: Nat Commun. 2019 Apr 5;10:1579. doi: 10.1038/s41467-019-09407-3 (PMC6450952; doi:10.1038/s41467-019-09407-3)
Supplement: Supplementary file 1 — Supplementary Information [file 41467_2019_9407_MOESM1_ESM.pdf]

**Supplementary Information for**

**Nguyen et al., “Phenotypes associated with genes encoding drug targets are predictive of clinical trial side effects”**

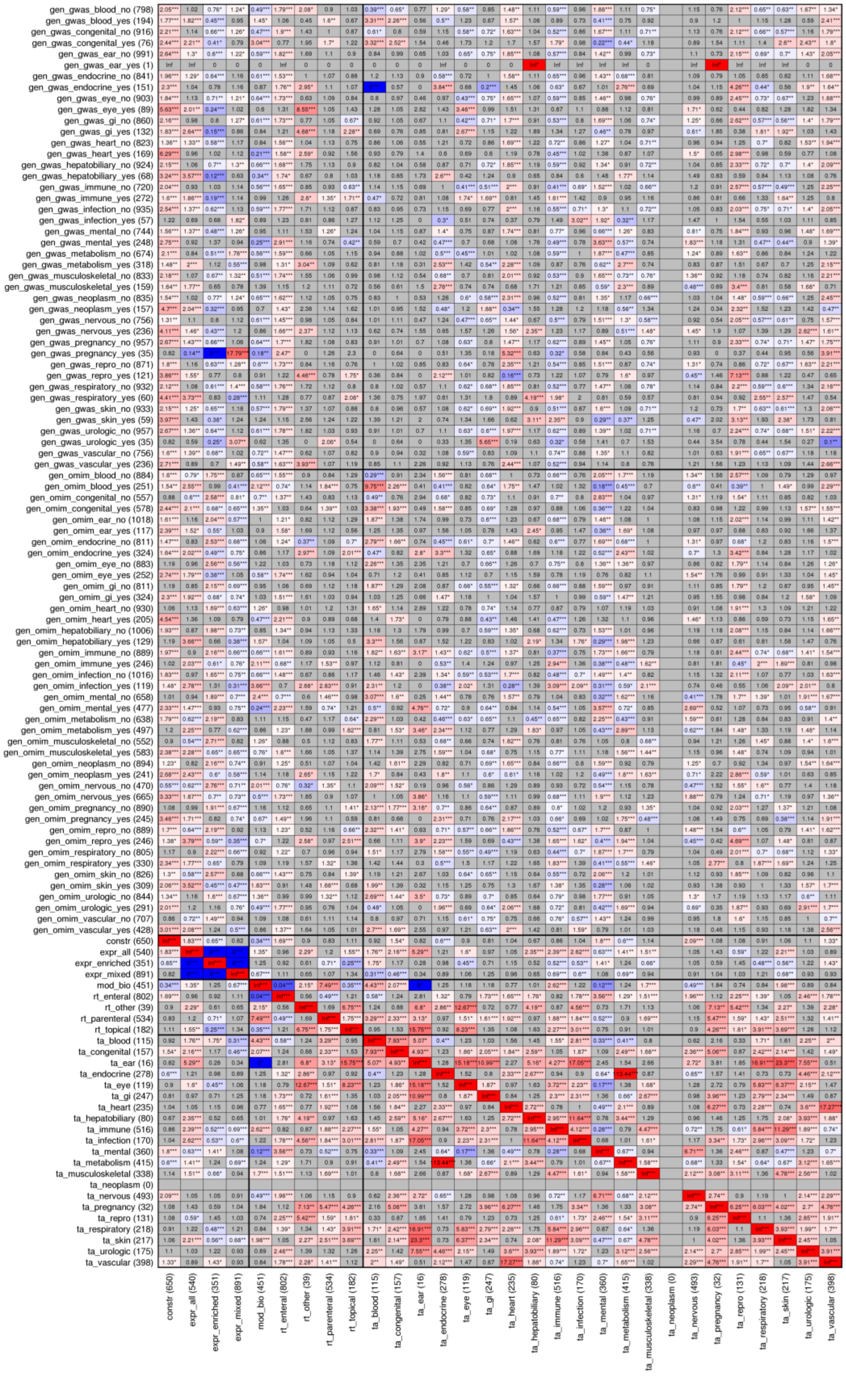

**<u>Supplementary Figure 1</u>**. Correlation of independent variables in side effect model. All variables are binary; correlations displayed are odds ratios (ORs); asterisks show P-value from Fisher's exact test (*, P < 0.05; **, P < 0.005; ***, P < 0.0005).

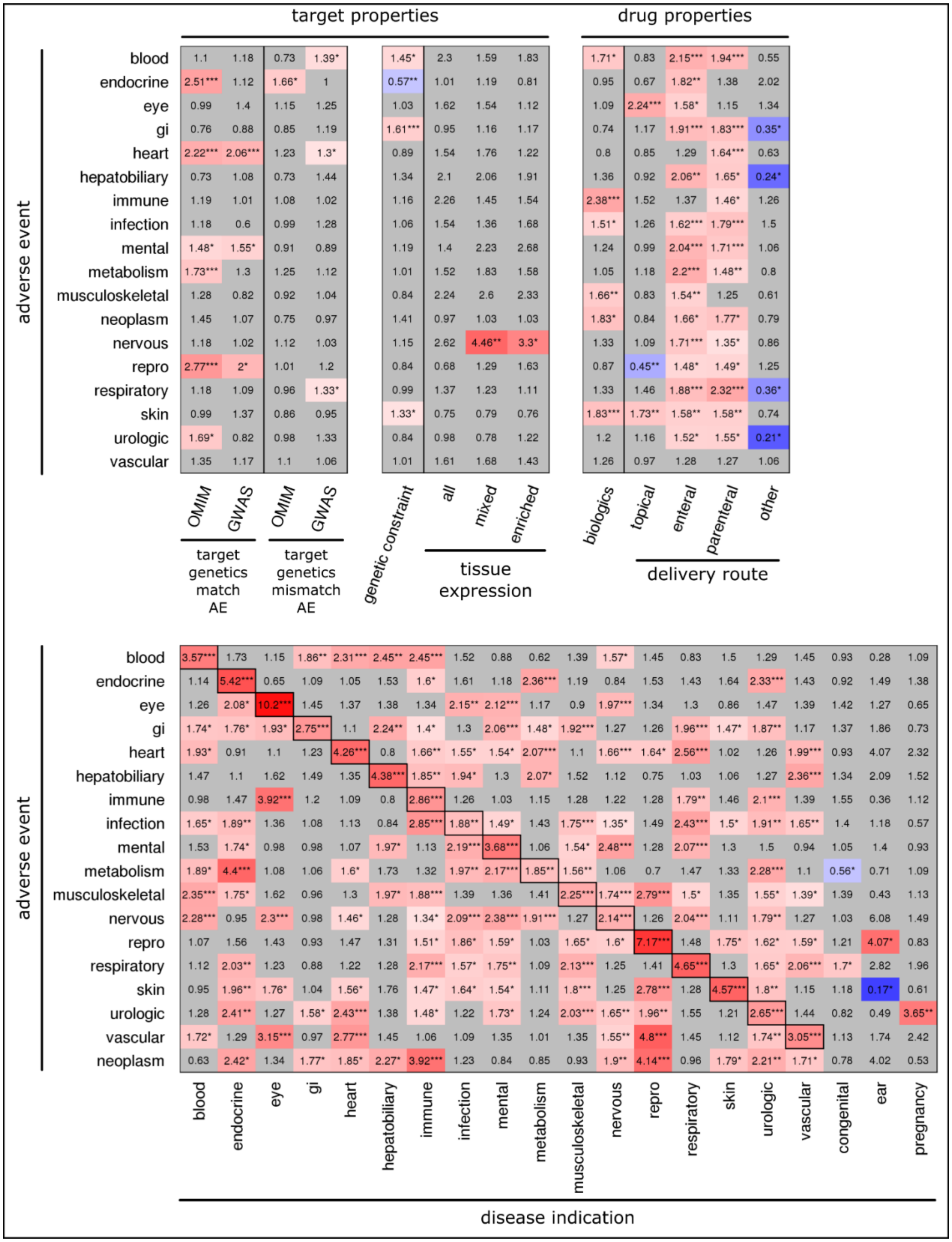


**Supplementary Figure 2**. All coefficients from each of the 18 side effect regression models, where “on-indication” side effects are retained in the data. Asterisks show P-value of the coefficient (*, P < 0.05; **, P < 0.005; ***, P < 0.0005).

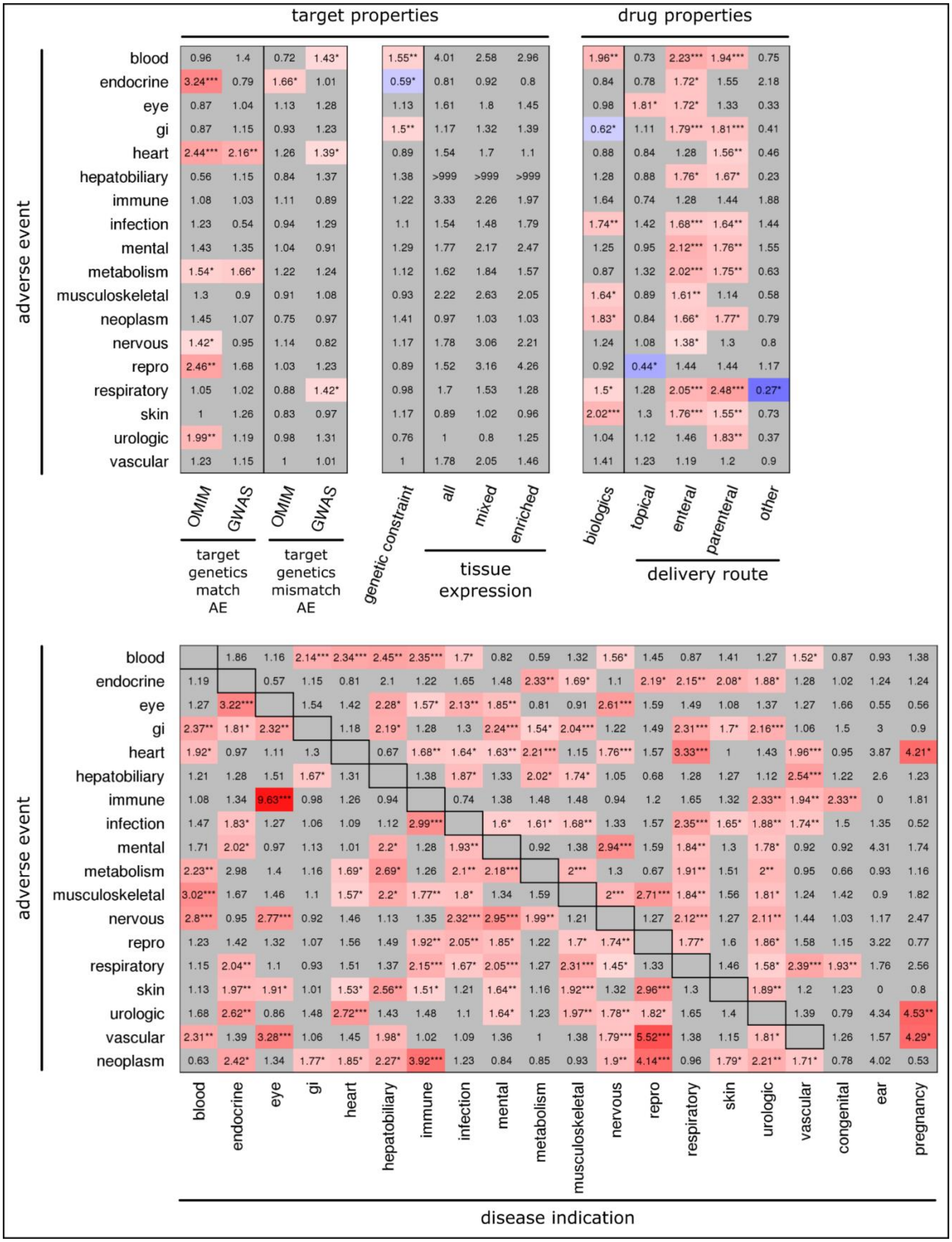


**Supplementary Figure 3**. All coefficients from each of the 18 side effect regression models, where only “off-indication” side effects are retained in the data. Asterisks show P-value of the coefficient (*, P < 0.05; **, P < 0.005; ***, P < 0.0005).

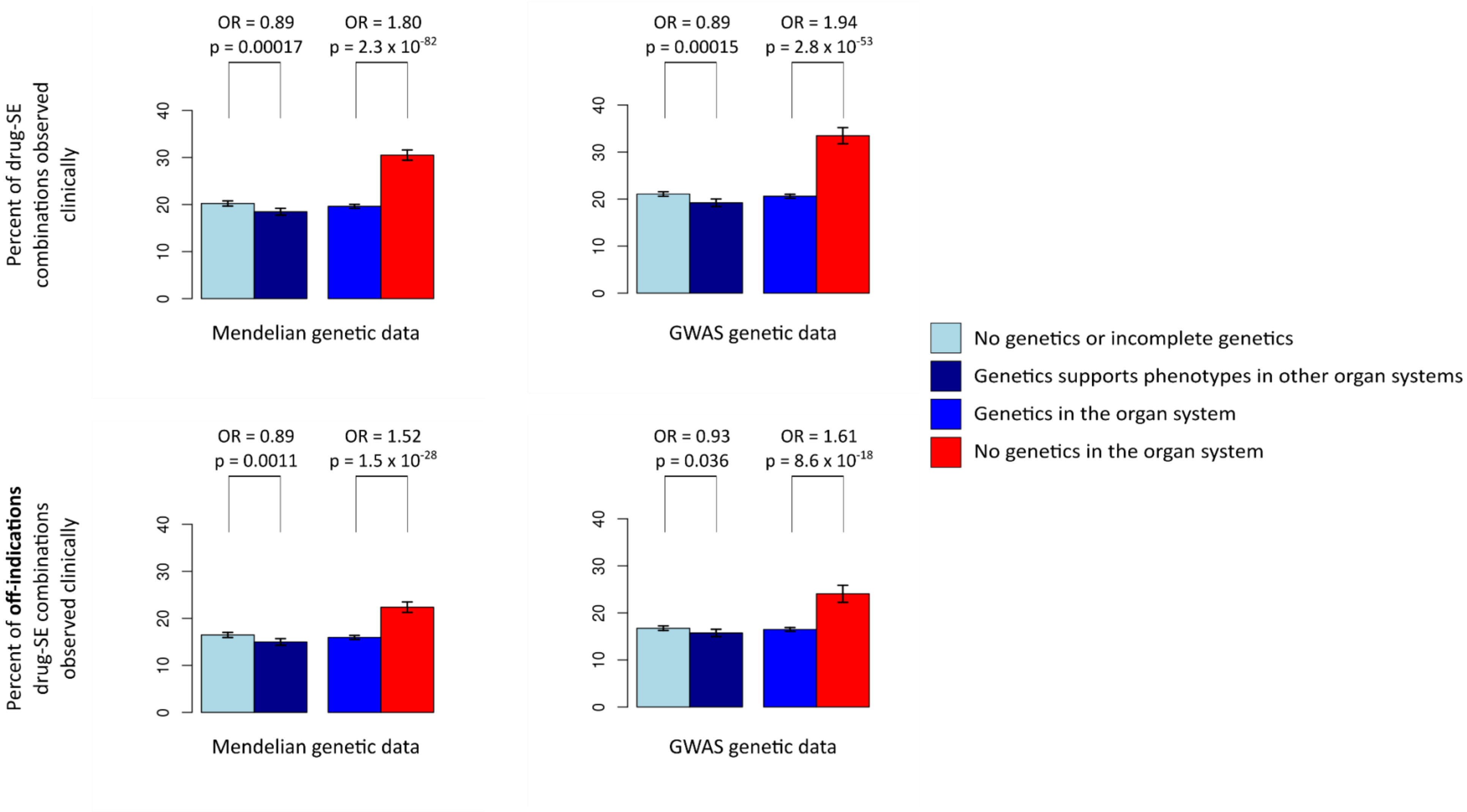


**Supplementary Figure 4**. Comparison of the rate at which all possible drug-SE (side effect) combinations are manifested as side effects in the clinical trial data set, between drug-SE pairs with or without genetic support. OR, odds ratio; P, P-value from Fisher's exact test (two-tailed). The top two panels show analyses based on all 38,199 drug-SE combinations; the bottom two panels show analyses based on 33,489 "off-indication" drug-SE combinations. The left two panels show analyses based on Mendelian genetic data; the right two panels show analyses based on GWAS genetic data. Error bars represent the 95% confidence interval of the reported proportions. Underlying data and confidence intervals for the OR values are shown in **Supplementary Table 1**.

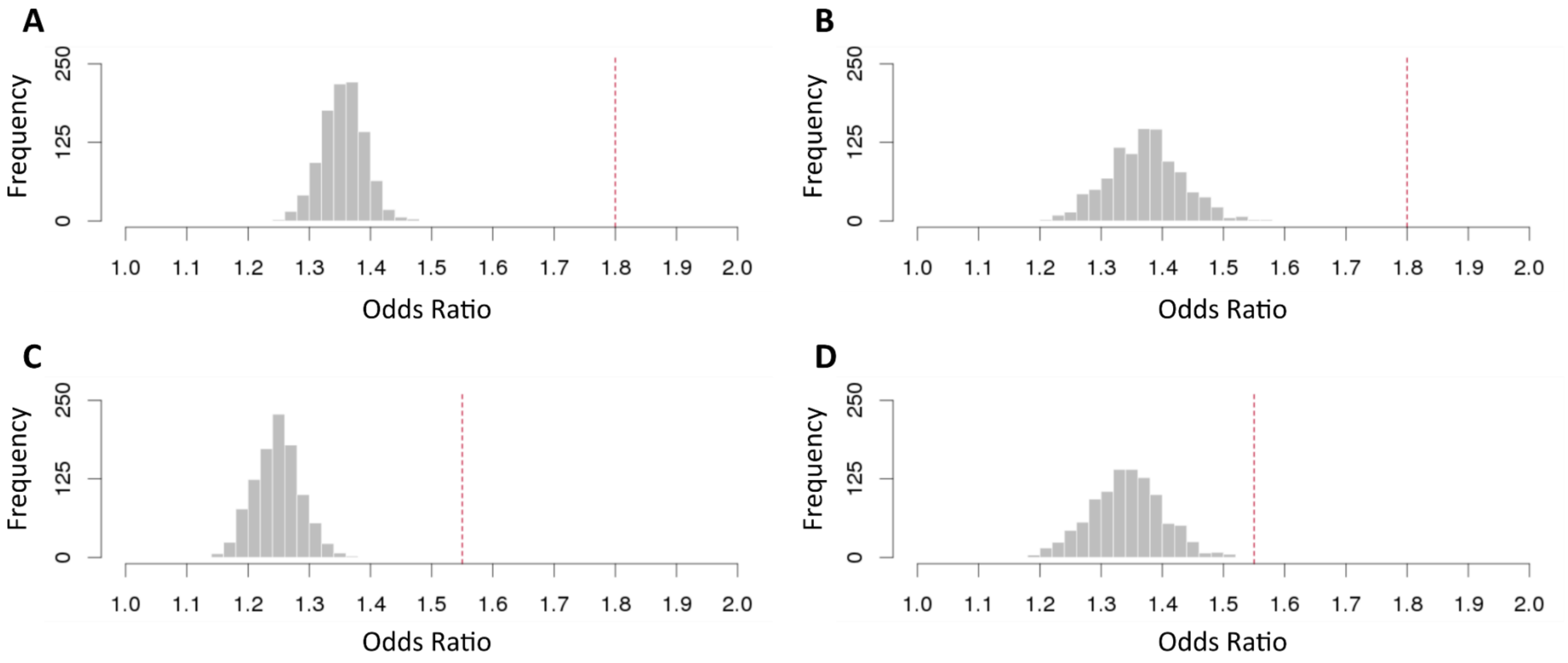


**<u>Supplementary Figure 5</u>**. Result of permutation test for significance of genetics-SE enrichment relative to randomized SEs and randomized target genetics. In each analysis, gray histogram shows ORs resulting from 1,000 simulations and dashed vertical line shows the OR of the study data. Mean and standard deviation of the simulation ORs are provided in **Supplementary Table 2**. In each analysis, the simulation OR never exceeds the study data OR (Monte Carlo empirical $P < 0.001$). Analyses used: (A) all drug-SE pairs, randomized SEs; (B) all drug-SE pairs, randomized target genetics; (C) only off-indication drug-SE pairs, randomized SEs; (D) only off-indication drug-SE pairs, randomized target genetics.

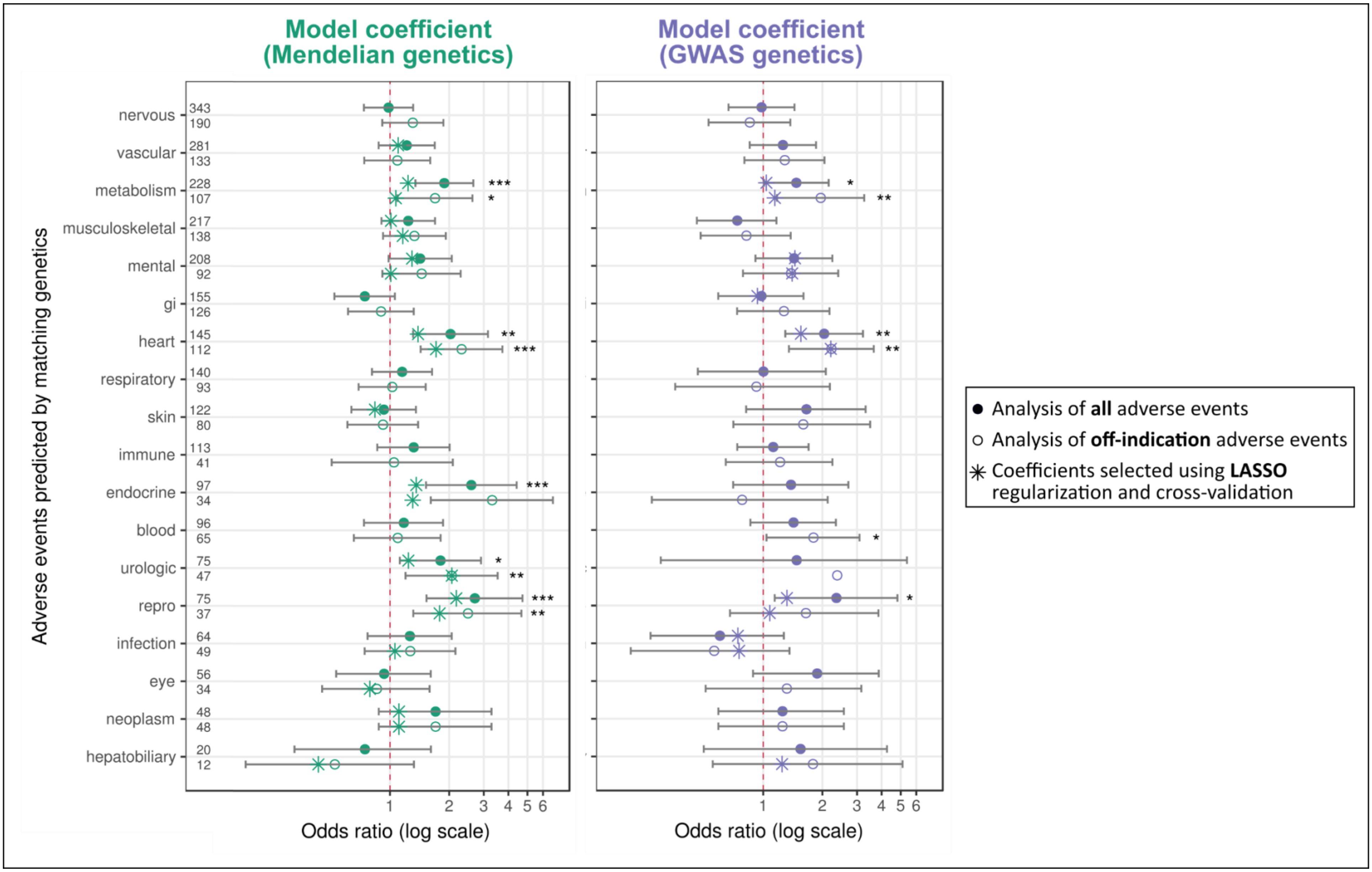


**Supplementary Figure 6**. Summary of side effect modeling results with quantitative, tissue-specific target gene expression values included in the models. LEFT: coefficients of the Mendelian genetics predictors in each of the 18 side effect models (from **Supplementary Table 9**). RIGHT: coefficients of the GWAS genetics predictors in each of the 18 side effect models (from **Supplementary Table 9**). Coefficients from the regression models were exponentiated to obtain odds ratios. Along each row, circular points indicate odds ratios from the glm models; error bars are 95% confidence intervals from the glm models; large eight-pointed stars indicate odds ratios when the genetics predictors are selected as predictors in the glmnet models (which are built using feature selection, lasso regularization, and cross-validation). Small asterisks offset from the data indicate P-values of the the Fisher's exact test (LEFT) and P-values of the coefficients from the glm models (CENTER and RIGHT); (*, P < 0.05; **, P < 0.01; ***, P < 0. 001).

| Data inclusion | Comparison | GS | GN | GS+GN | NS | NN | NS+NN | GS/GN | NS/NN | Odds Ratio | p | OR 95% CI | | GS/GS+GN | NS/NS+NN | stderr(GS/GS+GN) | sderr(NS/NS+NN) |
|---|---|---|---|---|---|---|---|---|---|---|---|---|---|---|---|---|---|
| All drug-SE combinations, all genetics | Overall: Genetic support vs no genetic support | 2512 | 5862 | 8374 | 5729 | 24096 | 29825 | 0.43 | 0.24 | 1.80 | 1.73E-94 | 1.71 | 1.90 | 30.00% | 19.21% | 0.50% | 0.23% |
| | Among no genetic support: incomplete genetic info vs complete genetic info | 2759 | 12165 | 14924 | 2970 | 11931 | 14901 | 0.23 | 0.25 | 0.91 | 0.00158 | 0.86 | 0.97 | 18.49% | 19.93% | 0.32% | 0.33% |
| All drug-SE combinations, Mendelian genetics | Overall: Genetic support vs no genetic support | 2092 | 4765 | 6857 | 6149 | 25193 | 31342 | 0.44 | 0.24 | 1.80 | 2.28E-82 | 1.70 | 1.91 | 30.51% | 19.62% | 0.56% | 0.22% |
| | Among no genetic support: incomplete genetic info vs complete genetic info | 2014 | 8893 | 10907 | 4135 | 16300 | 20435 | 0.23 | 0.25 | 0.89 | 0.00017 | 0.84 | 0.95 | 18.47% | 20.23% | 0.37% | 0.28% |
| All drug-SE combinations, GWAS genetics | Overall: Genetic support vs no genetic support | 962 | 1911 | 2873 | 7279 | 28047 | 35326 | 0.50 | 0.26 | 1.94 | 2.75E-53 | 1.79 | 2.11 | 33.48% | 20.61% | 0.88% | 0.22% |
| | Among no genetic support: incomplete genetic info vs complete genetic info | 1745 | 7334 | 9079 | 5534 | 20713 | 26247 | 0.24 | 0.27 | 0.89 | 0.00015 | 0.84 | 0.95 | 19.22% | 21.08% | 0.41% | 0.25% |
| Off-indication drug-SE combinations, all genetics | Overall: Genetic support vs no genetic support | 1458 | 5062 | 6520 | 4219 | 22750 | 26969 | 0.29 | 0.19 | 1.55 | 1.70E-36 | 1.45 | 1.66 | 22.36% | 15.64% | 0.52% | 0.22% |
| | Among no genetic support: incomplete genetic info vs complete genetic info | 2067 | 11595 | 13662 | 2152 | 11155 | 13307 | 0.18 | 0.19 | 0.92 | 0.01892 | 0.86 | 0.99 | 15.13% | 16.17% | 0.31% | 0.32% |
| Off-indication drug-SE combinations, Mendelian genetics | Overall: Genetic support vs no genetic support | 1180 | 4095 | 5275 | 4497 | 23717 | 28214 | 0.29 | 0.19 | 1.52 | 1.47E-28 | 1.41 | 1.63 | 22.37% | 15.94% | 0.57% | 0.22% |
| | Among no genetic support: incomplete genetic info vs complete genetic info | 1503 | 8528 | 10031 | 2994 | 15189 | 18183 | 0.18 | 0.20 | 0.89 | 0.00111 | 0.84 | 0.96 | 14.98% | 16.47% | 0.36% | 0.28% |
| Off-indication drug-SE combinations, GWAS genetics | Overall: Genetic support vs no genetic support | 506 | 1597 | 2103 | 5171 | 26215 | 31386 | 0.32 | 0.20 | 1.61 | 8.61E-18 | 1.44 | 1.78 | 24.06% | 16.48% | 0.93% | 0.21% |
| | Among no genetic support: incomplete genetic info vs complete genetic info | 1292 | 6919 | 8211 | 3879 | 19296 | 23175 | 0.19 | 0.20 | 0.93 | 0.0362 | 0.87 | 1.00 | 15.73% | 16.74% | 0.40% | 0.25% |

**Supplementary Table 1.** Comparison of the rate at which all possible drug-SE (side effect) combinations are manifested as side effects in the clinical trial data set, between drug-SE pairs with or without genetic support. OR, odds ratio; p, p-value from Fisher's exact test (two-tailed). GS = genetics + side effect; GN = genetics + no side effect; NS = no genetics + side effect; NN = no genetics + no side effect. For the overall comparison, "genetics" indicates a match between the side effect and genetic phenotype. For the "among no genetic support" comparison, "genetics" indicates that there is a genetic phenotype for the targets that mismatches; "no genetics" indicates there is no or incomplete genetic information for the target gene(s).

| Phenotype category | Main analysis (Drug x organ system) combinations | Drugs with genetics and side effect | Drugs with genetics and no side effect | Drugs with no genetics and side effect | Drugs with no genetics and no side effect | Rate of side effect when genetic association exists (precision) (PPV) | Rate of side effect when genetic association does not exist (FOR) | TPR (recall) (SEN) | TNR (SPE) | NPV | LR+ | iLR- | OR | 95% CI | | p |
|---|---|---|---|---|---|---|---|---|---|---|---|---|---|---|---|---|
| blood | | 96 | 269 | 234 | 1220 | 26.3% | 16.1% | 29.1% | 81.9% | 83.9% | 1.61 | 1.16 | 1.86 | 1.40 | 2.46 | 1.34E-05 |
| heart | | 145 | 176 | 451 | 1047 | 45.2% | 30.1% | 24.3% | 85.6% | 69.9% | 1.69 | 1.13 | 1.91 | 1.48 | 2.46 | 3.92E-07 |
| congenital | | 17 | 572 | 22 | 1208 | 2.9% | 1.8% | 43.6% | 67.9% | 98.2% | 1.36 | 1.20 | 1.63 | 0.81 | 3.25 | 0.165102913 |
| ear | | 5 | 112 | 74 | 1628 | 4.3% | 4.3% | 6.3% | 93.6% | 95.7% | 0.98 | 1.00 | 0.98 | 0.30 | 2.47 | 1 |
| endocrine | | 97 | 252 | 177 | 1293 | 27.8% | 12.0% | 35.4% | 83.7% | 88.0% | 2.17 | 1.30 | 2.81 | 2.09 | 3.76 | 3.71E-12 |
| eye | | 56 | 256 | 193 | 1314 | 17.9% | 12.8% | 22.5% | 83.7% | 87.2% | 1.38 | 1.08 | 1.49 | 1.05 | 2.08 | 0.018630562 |
| gi | | 155 | 276 | 497 | 891 | 36.0% | 35.8% | 23.8% | 76.3% | 64.2% | 1.01 | 1.00 | 1.01 | 0.80 | 1.27 | 0.95420768 |
| hepatobiliary | | 20 | 153 | 160 | 1486 | 11.6% | 9.7% | 11.1% | 90.7% | 90.3% | 1.19 | 1.02 | 1.21 | 0.70 | 2.01 | 0.42299002 |
| immune | | 113 | 335 | 251 | 1120 | 25.2% | 18.3% | 31.0% | 77.0% | 81.7% | 1.35 | 1.12 | 1.51 | 1.16 | 1.95 | 0.001757385 |
| infection | | 64 | 104 | 536 | 1115 | 38.1% | 32.5% | 10.7% | 91.5% | 67.5% | 1.25 | 1.02 | 1.28 | 0.91 | 1.80 | 0.143708732 |
| metabolism | | 228 | 400 | 280 | 911 | 36.3% | 23.5% | 44.9% | 69.5% | 76.5% | 1.47 | 1.26 | 1.85 | 1.49 | 2.30 | 1.34E-08 |
| musculoskeletal | | 217 | 435 | 322 | 845 | 33.3% | 27.6% | 40.3% | 66.0% | 72.4% | 1.18 | 1.11 | 1.31 | 1.06 | 1.62 | 0.01184709 |
| neoplasm | | 48 | 305 | 122 | 1344 | 13.6% | 8.3% | 28.2% | 81.5% | 91.7% | 1.53 | 1.14 | 1.73 | 1.19 | 2.50 | 0.003098258 |
| nervous | | 343 | 421 | 401 | 654 | 44.9% | 38.0% | 46.1% | 60.8% | 62.0% | 1.18 | 1.13 | 1.33 | 1.09 | 1.61 | 0.003732245 |
| pregnancy | | 7 | 268 | 35 | 1509 | 2.5% | 2.3% | 16.7% | 84.9% | 97.7% | 1.11 | 1.02 | 1.13 | 0.42 | 2.61 | 0.826676608 |
| mental | | 208 | 350 | 255 | 1006 | 37.3% | 20.2% | 44.9% | 74.2% | 79.8% | 1.74 | 1.35 | 2.34 | 1.87 | 2.94 | 5.16E-14 |
| urologic | | 75 | 245 | 220 | 1279 | 23.4% | 14.7% | 25.4% | 83.9% | 85.3% | 1.58 | 1.13 | 1.78 | 1.30 | 2.41 | 0.000219979 |
| repro | | 75 | 199 | 186 | 1359 | 27.4% | 12.0% | 28.7% | 87.2% | 88.0% | 2.25 | 1.22 | 2.75 | 2.00 | 3.77 | 5.75E-10 |
| respiratory | | 140 | 211 | 464 | 1004 | 39.9% | 31.6% | 23.2% | 82.6% | 68.4% | 1.33 | 1.08 | 1.44 | 1.12 | 1.84 | 0.003676008 |
| skin | | 122 | 224 | 413 | 1060 | 35.3% | 28.0% | 22.8% | 82.6% | 72.0% | 1.31 | 1.07 | 1.40 | 1.08 | 1.80 | 0.00874946 |
| vascular | | 281 | 299 | 436 | 803 | 48.4% | 35.2% | 39.2% | 72.9% | 64.8% | 1.44 | 1.20 | 1.73 | 1.41 | 2.13 | 8.31E-08 |
| **TOTAL** | **38199** | **2512** | **5862** | **5729** | **24096** | **30.0%** | **19.2%** | **30.5%** | **80.4%** | **80.8%** | **1.56** | **1.16** | **1.80** | **1.71** | **1.90** | **1.73E-94** |
| | | | | | | | | | | | | | | | | |
| | Main analysis with randomized data categories | | | | | | | | | | | | | | | |
| **Random genetics (mean of 1000 randomizations)** | **38199** | **2623** | **7615** | **5618** | **22343** | **25.6%** | **20.1%** | **31.8%** | **74.6%** | **79.9%** | **1.25** | **1.09** | **1.37** | **1.30** | **1.45** | **1.99687E-11** |
| Standard deviation of random genetics | | 121 | 334 | 121 | 334 | 0.58% | 0.22% | 1.46% | 1.12% | 0.22% | 0.04 | 0.02 | 0.06 | 0.06 | 0.06 | 6.11543E-10 |
| **Random side effects (mean of 1000 randomizations)** | **38199** | **2528** | **5846** | **7213** | **22612** | **30.2%** | **24.2%** | **26.0%** | **79.5%** | **75.8%** | **1.26** | **1.07** | **1.36** | **1.28** | **1.43** | **3.06273E-18** |
| Standard deviation of random side effects | | 47 | 47 | 109 | 109 | 0.6% | 0.4% | 0.4% | 0.1% | 0.37% | 0.02 | 0.01 | 0.03 | 0.03 | 0.04 | 8.94689E-17 |
| | | | | | | | | | | | | | | | | |
| | Main analysis using target annotations from single sources | | | | | | | | | | | | | | | |
| **DrugBank** | **15981** | **1402** | **2123** | **3788** | **8668** | **39.8%** | **30.4%** | **27.0%** | **80.3%** | **69.6%** | **1.37** | **1.10** | **1.51** | **1.40** | **1.63** | **4.28442E-25** |
| **Citeline Pharmaprojects** | **34944** | **1923** | **4480** | **5588** | **22953** | **30.0%** | **19.6%** | **25.6%** | **83.7%** | **80.4%** | **1.57** | **1.12** | **1.76** | **1.66** | **1.87** | **3.25909E-71** |
| **Santos et al (2017)** | **14133** | **1395** | **2012** | **3535** | **7191** | **40.9%** | **33.0%** | **28.3%** | **78.1%** | **67.0%** | **1.29** | **1.09** | **1.41** | **1.30** | **1.53** | **2.96E-17** |
| | | | | | | | | | | | | | | | | |
| | Replication analysis using OFFSIDES data | | | | | | | | | | | | | | | |
| **TOTAL** | **5523** | **1230** | **326** | **2940** | **1027** | **79.0%** | **74.1%** | **29.5%** | **75.9%** | **25.9%** | **1.22** | **1.08** | **1.32** | **1.14** | **1.52** | **0.000112548** |
| **Random genetics (mean of 1000 randomizations)** | **5523** | **1632** | **523** | **2538** | **830** | **75.7%** | **75.4%** | **39.1%** | **61.3%** | **24.6%** | **1.01** | **1.01** | **1.02** | **0.90** | **1.16** | **0.472879439** |
| **Standard deviation of random genetics** | | 85 | 35 | 85 | 35 | 0.8% | 0.5% | 2.0% | 2.6% | 0.5% | 0.04 | 0.03 | 0.07 | 0.06 | 0.08 | 0.301098261 |
| | | | | | | | | | | | | | | | | |
| | Analysis of placebo contribution to side effects using AACT data | | | | | | | | | | | | | | | |
| **Placebo-controlled side effects** | **4809** | **291** | **943** | **460** | **3115** | **23.6%** | **12.9%** | **38.7%** | **76.8%** | **87.1%** | **1.67** | **1.25** | **2.09** | **1.77** | **2.47** | **6.61954E-18** |
| **All placebo group side effects** | **4746** | **529** | **690** | **1151** | **2376** | **43.4%** | **32.6%** | **31.5%** | **77.5%** | **67.4%** | **1.40** | **1.13** | **1.58** | **1.38** | **1.81** | **2.32827E-11** |
| **All drug group side effects** | **4809** | **604** | **630** | **1232** | **2343** | **48.9%** | **34.5%** | **32.9%** | **78.8%** | **65.5%** | **1.55** | **1.17** | **1.82** | **1.60** | **2.08** | **4.12156E-19** |

| Phenotype category | Off-indication analysis (Drug x organ system) combinations | Drugs with genetics and side effect | Drugs with genetics and no side effect | Drugs with no genetics and side effect | Drugs with no genetics and no side effect | Rate of side effect when genetic association exists (precision) (PPV) | Rate of side effect when genetic association does not exist (FOR) | TPR (recall) (SEN) | TNR (SPE) | NPV | LR+ | iLR- | OR | 95% CI | | p |
|---|---|---|---|---|---|---|---|---|---|---|---|---|---|---|---|---|
| blood | | 65 | 234 | 212 | 1193 | 21.7% | 15.1% | 23.5% | 83.6% | 84.9% | 1.43 | 1.09 | 1.56 | 1.13 | 2.15 | 0.005724 |
| heart | | 112 | 162 | 318 | 992 | 40.9% | 24.3% | 26.0% | 86.0% | 75.7% | 1.86 | 1.16 | 2.16 | 1.63 | 2.85 | 5.90E-08 |
| congenital | | 9 | 507 | 11 | 1135 | 1.7% | 1.0% | 45.0% | 69.1% | 99.0% | 1.46 | 1.26 | 1.83 | 0.67 | 4.89 | 0.222399 |
| ear | | 4 | 112 | 65 | 1622 | 3.4% | 3.9% | 5.8% | 93.5% | 96.1% | 0.90 | 0.99 | 0.89 | 0.23 | 2.46 | 1 |
| endocrine | | 34 | 207 | 96 | 1204 | 14.1% | 7.4% | 26.2% | 85.3% | 92.6% | 1.78 | 1.16 | 2.06 | 1.31 | 3.17 | 0.001412 |
| eye | | 34 | 250 | 148 | 1268 | 12.0% | 10.5% | 18.7% | 83.5% | 89.5% | 1.13 | 1.03 | 1.17 | 0.76 | 1.75 | 0.4617 |
| gi | | 126 | 244 | 379 | 823 | 34.1% | 31.5% | 25.0% | 77.1% | 68.5% | 1.09 | 1.03 | 1.12 | 0.87 | 1.44 | 0.373119 |
| hepatobiliary | | 12 | 150 | 134 | 1443 | 7.4% | 8.5% | 8.2% | 90.6% | 91.5% | 0.87 | 0.99 | 0.86 | 0.42 | 1.60 | 0.766027 |
| immune | | 41 | 232 | 116 | 914 | 15.0% | 11.3% | 26.1% | 79.8% | 88.7% | 1.29 | 1.08 | 1.39 | 0.92 | 2.07 | 0.09472 |
| infection | | 49 | 89 | 455 | 1056 | 35.5% | 30.1% | 9.7% | 92.2% | 69.9% | 1.25 | 1.02 | 1.28 | 0.87 | 1.87 | 0.20933 |
| metabolism | | 107 | 292 | 185 | 820 | 26.8% | 18.4% | 36.6% | 73.7% | 81.6% | 1.40 | 1.16 | 1.62 | 1.22 | 2.15 | 0.0006 |
| musculoskeletal | | 138 | 373 | 221 | 749 | 27.0% | 22.8% | 38.4% | 66.8% | 77.2% | 1.16 | 1.08 | 1.25 | 0.97 | 1.61 | 0.07435 |
| neoplasm | | 48 | 305 | 122 | 1344 | 13.6% | 8.3% | 28.2% | 81.5% | 91.7% | 1.53 | 1.14 | 1.73 | 1.19 | 2.50 | 0.003098 |
| nervous | | 190 | 312 | 266 | 558 | 37.8% | 32.3% | 41.7% | 64.1% | 67.7% | 1.16 | 1.10 | 1.28 | 1.01 | 1.62 | 0.042694 |
| pregnancy | | 7 | 262 | 32 | 1486 | 2.6% | 2.1% | 17.9% | 85.0% | 97.9% | 1.20 | 1.04 | 1.24 | 0.46 | 2.90 | 0.648859 |
| mental | | 92 | 278 | 177 | 912 | 24.9% | 16.3% | 34.2% | 76.6% | 83.7% | 1.46 | 1.16 | 1.71 | 1.27 | 2.29 | 0.000342 |
| urologic | | 47 | 215 | 162 | 1220 | 17.9% | 11.7% | 22.5% | 85.0% | 88.3% | 1.50 | 1.10 | 1.65 | 1.13 | 2.37 | 0.008259 |
| repro | | 37 | 185 | 148 | 1318 | 16.7% | 10.1% | 20.0% | 87.7% | 89.9% | 1.62 | 1.10 | 1.78 | 1.17 | 2.66 | 0.005417 |
| respiratory | | 93 | 197 | 361 | 950 | 32.1% | 27.5% | 20.5% | 82.8% | 72.5% | 1.19 | 1.04 | 1.24 | 0.93 | 1.65 | 0.130584 |
| skin | | 80 | 211 | 307 | 1004 | 27.5% | 23.4% | 20.7% | 82.6% | 76.6% | 1.19 | 1.04 | 1.24 | 0.92 | 1.66 | 0.150424 |
| vascular | | 133 | 245 | 304 | 739 | 35.2% | 29.1% | 30.4% | 75.1% | 70.9% | 1.22 | 1.08 | 1.32 | 1.02 | 1.71 | 0.031836 |
| **TOTAL** | **33489** | **1458** | **5062** | **4219** | **22750** | **22.4%** | **15.6%** | **25.7%** | **81.8%** | **84.4%** | **1.41** | **1.10** | **1.55** | **1.45** | **1.66** | **1.70E-36** |
| | | | | | | | | | | | | | | | | |
| | Main analysis with randomized data categories | | | | | | | | | | | | | | | |
| **Random genetics (mean of 1000 randomizations)** | **33489** | **1762** | **6987** | **3915** | **20825** | **20.1%** | **15.8%** | **31.0%** | **74.9%** | **84.2%** | **1.24** | **1.09** | **1.34** | **1.26** | **1.43** | **2.78E-09** |
| Standard deviation of random genetics | | 85 | 317 | 85 | 317 | 0.5% | 0.2% | 1.5% | 1.1% | 0.2% | 0.04 | 0.01 | 0.06 | 0.06 | 0.06 | 6.41E-08 |
| **Random side effects (mean of 1000 randomizations)** | **33489** | **1705** | **4815** | **5964** | **21005** | **26.2%** | **22.1%** | **22.2%** | **81.4%** | **77.9%** | **1.19** | **1.05** | **1.25** | **1.17** | **1.33** | **2.53E-06** |
| Standard deviation of random side effects | | 37 | 37 | 100 | 100 | 0.6% | 0.4% | 0.4% | 0.1% | 0.4% | 0.03 | 0.01 | 0.04 | 0.04 | 0.04 | 6.96E-05 |
| | | | | | | | | | | | | | | | | |
| | Main analysis using target annotations from single sources | | | | | | | | | | | | | | | |
| **DrugBank** | **13438** | **755** | **1861** | **2694** | **8128** | **28.9%** | **24.9%** | **21.9%** | **81.4%** | **75.1%** | **1.17** | **1.04** | **1.22** | **1.11** | **1.35** | **3.84E-05** |
| **Citeline Pharmaprojects** | **30661** | **1073** | **3797** | **4108** | **21683** | **22.0%** | **15.9%** | **20.7%** | **85.1%** | **84.1%** | **1.39** | **1.07** | **1.49** | **1.38** | **1.61** | **3.83E-24** |
| **Santos et al (2017)** | **11770** | **765** | **1791** | **2481** | **6733** | **29.9%** | **26.9%** | **23.6%** | **79.0%** | **73.1%** | **1.12** | **1.03** | **1.16** | **1.05** | **1.28** | **0.002911** |
| | | | | | | | | | | | | | | | | |
| | Replication analysis using OFFSIDES data | | | | | | | | | | | | | | | |
| **TOTAL** | **4998** | **1003** | **317** | **2669** | **1009** | **76.0%** | **72.6%** | **27.3%** | **76.1%** | **27.4%** | **1.14** | **1.05** | **1.20** | **1.03** | **1.39** | **0.016452** |
| **Random genetics (mean of 1000 randomizations)** | **4998** | **1425** | **515** | **2247** | **811** | **73.5%** | **73.5%** | **38.8%** | **61.2%** | **26.5%** | **1.00** | **1.00** | **1.00** | **0.88** | **1.14** | **0.483419** |
| **Standard deviation of random genetics** | | 79 | 34 | 79 | 34 | 0.9% | 0.5% | 2.1% | 2.6% | 0.5% | 0.04 | 0.03 | 0.07 | 0.06 | 0.08 | 0.29747 |
| | | | | | | | | | | | | | | | | |
| | Analysis of placebo contribution to side effects using AACT data | | | | | | | | | | | | | | | |
| **Placebo-controlled side effects** | **4275** | **198** | **798** | **379** | **2900** | **19.9%** | **11.6%** | **34.3%** | **78.4%** | **88.4%** | **1.59** | **1.19** | **1.90** | **1.56** | **2.30** | **1.11E-10** |
| **All placebo group side effects** | **4041** | **330** | **590** | **910** | **2211** | **35.9%** | **29.2%** | **26.6%** | **78.9%** | **70.8%** | **1.26** | **1.08** | **1.36** | **1.16** | **1.59** | **0.00013** |
| **All drug group side effects** | **4091** | **386** | **544** | **966** | **2195** | **41.5%** | **30.6%** | **28.6%** | **80.1%** | **69.4%** | **1.44** | **1.12** | **1.61** | **1.38** | **1.88** | **7.35E-10** |

**Supplementary Table 2.** Analysis of enrichment between genetic phenotypes and side effects in individual organ systems. P value is from Fisher's exact test (two-sided.) PPV, positive predictive value; FOR, false omission rate; TPR, true positive rate; TNR, true negative rate; SEN, sensitivity; SPE, specificity; PPV, positive predictive value; NPV, negative predictive value; LR+, likelihood ratio positive (SEN/1-SPE); iLR-, inverse likelihood ratio negative (SPE/1-SEN)

| | | Model: glm (all data used) | | | | | | | | Model: glm (only off-indication side effects used) | | | | | | | | Model: glmnet (all data used) | | Model:glmnet (only off-indication data used) | |
|---|---|---|---|---|---|---|---|---|---|---|---|---|---|---|---|---|---|---|---|---|---|
| Model (Side effect predicted) | Variable | beta | 95% CI | | OR | 95% CI | | p | | beta | 95% CI | | OR | 95% CI | | p | | beta | OR | beta | OR |
| blood | gen_omim_blood_yes | 0.099252 | -0.34305 | 0.53315 | 1.104344 | 0.709606 | 1.704292 | 0.656639 | | -0.04189 | -0.53206 | 0.433955 | 0.95898 | 0.587394 | 1.5433489 | 0.86482 | | NA | NA | NA | NA |
| heart | gen_omim_heart_yes | 0.798367 | 0.40002 | 1.196922 | 2.221909 | 1.491855 | 3.309912 | 8.48E-05 | | 0.893515 | 0.461857 | 1.324631 | 2.443704 | 1.587018 | 3.7607955 | 4.83E-05 | | 0.379982 | 1.462257677 | 0.443476494 | 1.55811459 |
| congenital | gen_omim_congenital_yes | 0.621939 | -0.41148 | 1.675398 | 1.862537 | 0.662668 | 5.340923 | 0.238712 | | 1.426093 | -0.03082 | 2.992485 | 4.162406 | 0.969653 | 19.935167 | 0.061713 | | NA | NA | NA | NA |
| ear | gen_omim_ear_yes | 0.103855 | -1.12003 | 1.144788 | 1.10944 | 0.32627 | 3.141776 | 0.855014 | | -0.05183 | -1.37712 | 1.042855 | 0.949494 | 0.252304 | 2.8373059 | 0.931646 | | NA | NA | NA | NA |
| endocrine | gen_omim_endocrine_yes | 0.920205 | 0.433658 | 1.405786 | 2.509804 | 1.542892 | 4.07873 | 0.000202 | | 1.176142 | 0.520462 | 1.824507 | 3.241844 | 1.682805 | 6.1997361 | 0.000389 | | 0.402171 | 1.495067668 | 0.517774649 | 1.678288709 |
| eye | gen_omim_eye_yes | -0.01432 | -0.54349 | 0.498809 | 0.985786 | 0.580716 | 1.646759 | 0.956975 | | -0.14153 | -0.73845 | 0.430976 | 0.868025 | 0.477852 | 1.538758 | 0.634138 | | NA | NA | NA | NA |
| gi | gen_omim_gi_yes | -0.2791 | -0.61287 | 0.050886 | 0.756466 | 0.541792 | 1.052203 | 0.099073 | | -0.13512 | -0.49874 | 0.224737 | 0.873614 | 0.607296 | 1.2519929 | 0.463764 | | NA | NA | NA | NA |
| hepatobiliary | gen_omim_hepatobiliary_yes | -0.31603 | -1.08987 | 0.398177 | 0.729037 | 0.336262 | 1.489108 | 0.403114 | | -0.58539 | -1.54517 | 0.254625 | 0.556886 | 0.213276 | 1.2899775 | 0.19804 | | NA | NA | -1.4535634 | 0.233735908 |
| immune | gen_omim_immune_yes | 0.173179 | -0.23424 | 0.57302 | 1.189079 | 0.791174 | 1.773615 | 0.399819 | | 0.081383 | -0.61842 | 0.739655 | 1.084786 | 0.538796 | 2.0952133 | 0.813348 | | NA | NA | NA | NA |
| infection | gen_omim_infection_yes | 0.166921 | -0.30967 | 0.637908 | 1.181661 | 0.733687 | 1.892518 | 0.489208 | | 0.208626 | -0.3043 | 0.715541 | 1.231985 | 0.73764 | 2.0452932 | 0.42181 | | NA | NA | 0.097292406 | 1.102182611 |
| metabolism | gen_omim_metabolism_yes | 0.546033 | 0.226133 | 0.866761 | 1.726391 | 1.253742 | 2.379191 | 0.000827 | | 0.433503 | 0.019678 | 0.845068 | 1.542653 | 1.019873 | 2.3281362 | 0.039288 | | 0.274074 | 1.315311652 | 0.107500297 | 1.113491191 |
| musculoskeletal | gen_omim_musculoskeletal_yes | 0.249683 | -0.04309 | 0.542835 | 1.283618 | 0.957828 | 1.720878 | 0.094643 | | 0.262713 | -0.08025 | 0.606263 | 1.300453 | 0.922887 | 1.8335675 | 0.133269 | | 0.189056 | 1.208108824 | 0.206192739 | 1.228990055 |
| neoplasm | gen_omim_neoplasm_yes | 0.369504 | -0.21864 | 0.94674 | 1.447017 | 0.803608 | 2.577293 | 0.213025 | | 0.369504 | -0.21864 | 0.94674 | 1.447017 | 0.803608 | 2.5772932 | 0.213025 | | 0.107302 | 1.113270488 | 0.116158947 | 1.123174384 |
| nervous | gen_omim_nervous_yes | 0.164469 | -0.09983 | 0.429047 | 1.178767 | 0.904993 | 1.535794 | 0.222629 | | 0.35222 | 0.029312 | 0.676456 | 1.422222 | 1.029745 | 1.9668946 | 0.032763 | | NA | NA | 0.001909478 | 1.001911303 |
| pregnancy | gen_omim_pregnancy_yes | 0.421696 | -0.70824 | 1.493486 | 1.524545 | 0.492511 | 4.452591 | 0.447656 | | 0.61356 | -0.56868 | 1.75126 | 1.846995 | 0.566272 | 5.7618578 | 0.294489 | | 0.431174 | 1.539063673 | 0.235694415 | 1.265787446 |
| mental | gen_omim_mental_yes | 0.394375 | 0.056778 | 0.731652 | 1.483456 | 1.058421 | 2.078512 | 0.021885 | | 0.354422 | -0.06954 | 0.775564 | 1.425356 | 0.932819 | 2.1718161 | 0.099758 | | 0.242969 | 1.27502867 | NA | NA |
| urologic | gen_omim_urologic_yes | 0.523558 | 0.086856 | 0.957154 | 1.688023 | 1.09074 | 2.604275 | 0.01822 | | 0.686187 | 0.190969 | 1.176972 | 1.986129 | 1.210422 | 3.2445342 | 0.006277 | | 0.319198 | 1.376023453 | 0.676594242 | 1.967166618 |
| repro | gen_omim_repro_yes | 1.018365 | 0.486548 | 1.542835 | 2.768663 | 1.626692 | 4.677832 | 0.000153 | | 0.900936 | 0.281338 | 1.503347 | 2.461906 | 1.324901 | 4.4967137 | 0.003764 | | 0.735977 | 2.087520169 | 0.553897702 | 1.740021904 |
| respiratory | gen_omim_respiratory_yes | 0.162156 | -0.17579 | 0.498038 | 1.176044 | 0.838793 | 1.645489 | 0.345175 | | 0.049954 | -0.32414 | 0.420287 | 1.051223 | 0.723149 | 1.522399 | 0.792341 | | NA | NA | NA | NA |
| skin | gen_omim_skin_yes | -0.0127 | -0.37601 | 0.346651 | 0.987381 | 0.686597 | 1.414324 | 0.945036 | | 0.002884 | -0.39431 | 0.394377 | 1.002888 | 0.674148 | 1.4834594 | 0.988552 | | NA | NA | NA | NA |
| vascular | gen_omim_vascular_yes | 0.296978 | -0.00848 | 0.601999 | 1.345786 | 0.99156 | 1.825765 | 0.056386 | | 0.205072 | -0.15162 | 0.559345 | 1.227613 | 0.859311 | 1.7495254 | 0.257793 | | 0.062813 | 1.064827403 | NA | NA |
| blood | gen_omim_blood_no | -0.30922 | -0.63336 | 0.014408 | 0.734019 | 0.530805 | 1.014512 | 0.061078 | | -0.33352 | -0.67043 | 0.002645 | 0.716398 | 0.51149 | 1.0026486 | 0.051854 | | NA | NA | NA | NA |
| heart | gen_omim_heart_no | 0.209043 | -0.05175 | 0.471349 | 1.232498 | 0.949566 | 1.602154 | 0.117013 | | 0.229932 | -0.05484 | 0.51703 | 1.258514 | 0.946638 | 1.6770403 | 0.11474 | | NA | NA | NA | NA |
| congenital | gen_omim_congenital_no | 0.174663 | -0.85064 | 1.183732 | 1.190845 | 0.427142 | 3.266544 | 0.733438 | | -0.8046 | -2.77941 | 0.773803 | 0.447269 | 0.062075 | 2.1679949 | 0.358564 | | NA | NA | NA | NA |
| ear | gen_omim_ear_no | 0.303676 | -0.26813 | 0.896471 | 1.35483 | 0.764805 | 2.450939 | 0.304859 | | 0.341274 | -0.24694 | 0.953078 | 1.406739 | 0.781185 | 2.5936797 | 0.262961 | | NA | NA | NA | NA |
| endocrine | gen_omim_endocrine_no | 0.507616 | 0.121747 | 0.899541 | 1.661325 | 1.129468 | 2.458475 | 0.010412 | | 0.50697 | 0.018401 | 1.010406 | 1.660253 | 1.018572 | 2.7467165 | 0.044543 | | NA | NA | NA | NA |
| eye | gen_omim_eye_no | 0.14363 | -0.21984 | 0.510929 | 1.154457 | 0.802647 | 1.666839 | 0.440448 | | 0.119536 | -0.28465 | 0.529013 | 1.126974 | 0.752274 | 1.697256 | 0.564026 | | NA | NA | NA | NA |
| gi | gen_omim_gi_no | -0.16726 | -0.42457 | 0.089663 | 0.845983 | 0.654051 | 1.093805 | 0.202097 | | -0.07373 | -0.35468 | 0.207464 | 0.928925 | 0.701401 | 1.2305536 | 0.606949 | | NA | NA | NA | NA |
| hepatobiliary | gen_omim_hepatobiliary_no | -0.31032 | -0.70888 | 0.089617 | 0.733215 | 0.492193 | 1.093755 | 0.127056 | | -0.17617 | -0.5964 | 0.248737 | 0.838472 | 0.550791 | 1.2824051 | 0.412964 | | NA | NA | NA | NA |
| immune | gen_omim_immune_no | 0.076126 | -0.24497 | 0.398987 | 1.079098 | 0.782727 | 1.490314 | 0.642807 | | 0.103422 | -0.34213 | 0.557787 | 1.108959 | 0.710258 | 1.7468024 | 0.65176 | | NA | NA | NA | NA |
| infection | gen_omim_infection_no | -0.01469 | -0.27189 | 0.243534 | 0.985421 | 0.761937 | 1.275749 | 0.911021 | | -0.06175 | -0.33389 | 0.211443 | 0.94012 | 0.716135 | 1.2354594 | 0.656951 | | NA | NA | NA | NA |
| metabolism | gen_omim_metabolism_no | 0.222239 | -0.08712 | 0.532415 | 1.24887 | 0.916571 | 1.703041 | 0.159371 | | 0.201384 | -0.15832 | 0.562911 | 1.223094 | 0.853574 | 1.7557755 | 0.273223 | | NA | NA | NA | NA |
| musculoskeletal | gen_omim_musculoskeletal_no | -0.08862 | -0.38384 | 0.204997 | 0.915197 | 0.681241 | 1.227521 | 0.554984 | | -0.08956 | -0.42875 | 0.24736 | 0.914335 | 0.651326 | 1.2806406 | 0.603254 | | NA | NA | NA | NA |
| neoplasm | gen_omim_neoplasm_no | -0.28779 | -0.7287 | 0.151101 | 0.74992 | 0.482537 | 1.163114 | 0.19889 | | -0.28779 | -0.7287 | 0.151101 | 0.74992 | 0.482537 | 1.1631137 | 0.19889 | | NA | NA | NA | NA |
| nervous | gen_omim_nervous_no | 0.11666 | -0.16462 | 0.397413 | 1.123737 | 0.848214 | 1.487971 | 0.41562 | | 0.134306 | -0.18593 | 0.454024 | 1.143742 | 0.830329 | 1.5746365 | 0.410373 | | NA | NA | NA | NA |
| pregnancy | gen_omim_pregnancy_no | 0.030128 | -0.72425 | 0.803572 | 1.030586 | 0.484686 | 2.233505 | 0.93792 | | 0.070496 | -0.7275 | 0.895624 | 1.07304 | 0.483116 | 2.4488642 | 0.863672 | | NA | NA | NA | NA |
| mental | gen_omim_mental_no | -0.09919 | -0.41892 | 0.2194 | 0.905569 | 0.657758 | 1.24533 | 0.542113 | | 0.03848 | -0.32143 | 0.39865 | 1.03923 | 0.725115 | 1.4898116 | 0.833887 | | NA | NA | NA | NA |
| urologic | gen_omim_urologic_no | -0.02075 | -0.37194 | 0.332309 | 0.979466 | 0.689398 | 1.394184 | 0.90795 | | -0.02529 | -0.4146 | 0.367039 | 0.975028 | 0.660607 | 1.4434536 | 0.898916 | | NA | NA | NA | NA |
| repro | gen_omim_repro_no | 0.005603 | -0.37212 | 0.386664 | 1.005619 | 0.689268 | 1.472061 | 0.976875 | | 0.028488 | -0.38166 | 0.44305 | 1.028897 | 0.682728 | 1.5574506 | 0.89211 | | NA | NA | NA | NA |
| respiratory | gen_omim_respiratory_no | -0.04264 | -0.31978 | 0.234755 | 0.958256 | 0.726308 | 1.264599 | 0.762962 | | -0.12972 | -0.42998 | 0.170356 | 0.87834 | 0.650521 | 1.1857275 | 0.396656 | | NA | NA | NA | NA |
| skin | gen_omim_skin_no | -0.14825 | -0.42036 | 0.123786 | 0.862217 | 0.656809 | 1.131773 | 0.285255 | | -0.18289 | -0.48146 | 0.11543 | 0.832861 | 0.617882 | 1.1223559 | 0.22938 | | NA | NA | NA | NA |
| vascular | gen_omim_vascular_no | 0.092819 | -0.16625 | 0.352135 | 1.097263 | 0.846832 | 1.4221 | 0.482538 | | 0.002697 | -0.28467 | 0.289839 | 1.0027 | 0.752264 | 1.3362127 | 0.98531 | | NA | NA | NA | NA |
| blood | gen_gwas_blood_yes | 0.162028 | -0.31809 | 0.630367 | 1.175893 | 0.72754 | 1.8783 | 0.502461 | | 0.336192 | -0.185 | 0.842622 | 1.399608 | 0.8311 | 2.3224477 | 0.19882 | | NA | NA | NA | NA |
| heart | gen_gwas_heart_yes | 0.724534 | 0.298394 | 1.149306 | 2.063768 | 1.347692 | 3.156001 | 0.000835 | | 0.772248 | 0.307414 | 1.233709 | 2.164627 | 1.359904 | 3.4339432 | 0.001068 | | 0.463002 | 1.588836587 | 0.487335477 | 1.627972665 |
| congenital | gen_gwas_congenital_yes | 0.569231 | -1.00026 | 1.974004 | 1.766908 | 0.367784 | 7.199449 | 0.445803 | | -20.0239 | NA | 233.7091 | 2.01E-09 | NA | 3.15E+101 | 0.996595 | | NA | NA | NA | NA |
| ear | gen_gwas_ear_yes | -14.8072 | NA | 93.63155 | 3.71E-07 | NA | 4.61E+40 | 0.977937 | | -14.8801 | NA | 93.5294 | 3.45E-07 | NA | 4.16E+40 | 0.977828 | | NA | NA | -0.448159095 | 0.638803046 |
| endocrine | gen_gwas_endocrine_yes | 0.115439 | -0.48692 | 0.713419 | 1.122366 | 0.614513 | 2.040958 | 0.705844 | | -0.23799 | -1.16635 | 0.637846 | 0.788211 | 0.311503 | 1.8924005 | 0.603265 | | NA | NA | NA | NA |
| eye | gen_gwas_eye_yes | 0.334411 | -0.38748 | 1.022525 | 1.397117 | 0.678767 | 2.780205 | 0.351392 | | 0.038179 | -0.8748 | 0.868451 | 1.038917 | 0.416947 | 2.3832159 | 0.93119 | | NA | NA | NA | NA |
| gi | gen_gwas_gi_yes | -0.12771 | -0.60508 | 0.335362 | 0.880105 | 0.546032 | 1.398447 | 0.593831 | | 0.135538 | -0.37781 | 0.633118 | 1.145153 | 0.685358 | 1.8834745 | 0.598429 | | NA | NA | NA | NA |
| hepatobiliary | gen_gwas_hepatobiliary_yes | 0.075288 | -1.02472 | 1.039194 | 1.078194 | 0.358898 | 2.826938 | 0.885083 | | 0.140888 | -0.97956 | 1.121122 | 1.151296 | 0.375475 | 3.0682958 | 0.790253 | | NA | NA | 0.528095345 | 1.695699508 |
| immune | gen_gwas_immune_yes | 0.0117 | -0.39377 | 0.407634 | 1.011769 | 0.674512 | 1.503257 | 0.954304 | | 0.025344 | -0.57959 | 0.600225 | 1.025668 | 0.56013 | 1.8225293 | 0.932645 | | NA | NA | NA | NA |
| infection | gen_gwas_infection_yes | -0.51125 | -1.30461 | 0.211812 | 0.599748 | 0.271278 | 1.235916 | 0.183923 | | -0.60729 | -1.55772 | 0.237838 | 0.544827 | 0.210615 | 1.2685041 | 0.181764 | | -0.29694 | 0.743089249 | -0.451750667 | 0.636512854 |
| metabolism | gen_gwas_metabolism_yes | 0.262958 | -0.09483 | 0.618067 | 1.300772 | 0.909524 | 1.855339 | 0.147905 | | 0.50413 | 0.032969 | 0.968735 | 1.655544 | 1.033519 | 2.6346094 | 0.034466 | | 0.09425 | 1.098834273 | 0.159039195 | 1.172383898 |
| musculoskeletal | gen_gwas_musculoskeletal_yes | -0.20233 | -0.65468 | 0.236152 | 0.816826 | 0.51961 | 1.266366 | 0.37262 | | -0.10698 | -0.62214 | 0.389184 | 0.898545 | 0.536796 | 1.4757755 | 0.67781 | | -0.09985 | 0.904974139 | NA | NA |
| neoplasm | gen_gwas_neoplasm_yes | 0.069713 | -0.63759 | 0.739822 | 1.072201 | 0.528563 | 2.095562 | 0.842241 | | 0.069713 | -0.63759 | 0.739822 | 1.072201 | 0.528563 | 2.0955617 | 0.842241 | | NA | NA | NA | NA |
| nervous | gen_gwas_nervous_yes | 0.023392 | -0.33625 | 0.379993 | 1.023668 | 0.714447 | 1.462274 | 0.898046 | | -0.05043 | -0.4853 | 0.377362 | 0.950818 | 0.615516 | 1.4584324 | 0.818508 | | NA | NA | NA | NA |
| pregnancy | gen_gwas_pregnancy_yes | -13.2782 | -211.482 | 15.82898 | 1.71E-06 | 1.43E-92 | 7489241 | 0.983308 | | -13.9709 | -361.641 | 29.85508 | 8.56E-07 | 8.74E-158 | 9.245E+12 | 0.989312 | | -0.10241 | 0.902663585 | NA | NA |
| mental | gen_gwas_mental_yes | 0.437294 | 0.040021 | 0.832048 | 1.548512 | 1.040833 | 2.29802 | 0.030312 | | 0.301085 | -0.21169 | 0.803571 | 1.351324 | 0.809219 | 2.2335026 | 0.244285 | | 0.354446 | 1.425390886 | 0.161250944 | 1.174979786 |
| urologic | gen_gwas_urologic_yes | -0.19243 | -1.68314 | 0.982415 | 0.824955 | 0.185789 | 2.670899 | 0.772721 | | 0.175497 | -1.20196 | 1.295228 | 1.191838 | 0.300604 | 3.6518277 | 0.779012 | | NA | NA | NA | NA |
| repro | gen_gwas_repro_yes | 0.693758 | 0.041341 | 1.333841 | 2.001223 | 1.042207 | 3.795593 | 0.035124 | | 0.51693 | -0.30272 | 1.290003 | 1.676871 | 0.738806 | 3.6327963 | 0.201307 | | 0.245466 | 1.278216898 | 0.040778328 | 1.041621182 |
| respiratory | gen_gwas_respiratory_yes | 0.088458 | -0.64665 | 0.788274 | 1.092488 | 0.523798 | 2.199597 | 0.808494 | | 0.015078 | -0.90957 | 0.847273 | 1.015193 | 0.402696 | 2.3332745 | 0.972961 | | NA | NA | NA | NA |
| skin | gen_gwas_skin_yes | 0.31313 | -0.35707 | 0.969059 | 1.367699 | 0.699723 | 2.635463 | 0.353218 | | 0.231386 | -0.55479 | 0.978249 | 1.260346 | 0.574194 | 2.6597953 | 0.552149 | | NA | NA | NA | NA |
| vascular | gen_gwas_vascular_yes | 0.156618 | -0.2055 | 0.516452 | 1.169549 | 0.814242 | 1.67607 | 0.394734 | | 0.143537 | -0.29822 | 0.576597 | 1.15435 | 0.742137 | 1.7799706 | 0.519491 | | NA | NA | NA | NA |
| blood | gen_gwas_blood_no | 0.330367 | 0.012208 | 0.650592 | 1.391478 | 1.012283 | 1.916676 | 0.042318 | | 0.36034 | 0.025948 | 0.697693 | 1.433817 | 1.026288 | 2.0091128 | 0.035302 | | NA | NA | NA | NA |
| heart | gen_gwas_heart_no | 0.265152 | 0.007513 | 0.523299 | 1.30363 | 1.007542 | 1.687586 | 0.043771 | | 0.331716 | 0.050142 | 0.614512 | 1.393357 | 1.05142 | 1.8487546 | 0.021134 | | NA | NA | NA | NA |
| congenital | gen_gwas_congenital_no | 0.412451 | -0.45215 | 1.298133 | 1.510516 | 0.636258 | 3.662451 | 0.352042 | | -0.45341 | -1.79347 | 0.859144 | 0.635459 | 0.166382 | 2.3611392 | 0.498311 | | NA | NA | NA | NA |
| ear | gen_gwas_ear_no | -0.20717 | -0.77257 | 0.35721 | 0.812882 | 0.461826 | 1.429336 | 0.470948 | | -0.15516 | -0.73549 | 0.425886 | 0.856281 | 0.479269 | 1.5309455 | 0.59932 | | NA | NA | NA | NA |
| endocrine | gen_gwas_endocrine_no | -0.00306 | -0.35738 | 0.351539 | 0.996944 | 0.699506 | 1.421253 | 0.986481 | | 0.010078 | -0.43665 | 0.458916 | 1.010129 | 0.646199 | 1.582357 | 0.964744 | | NA | NA | NA | NA |
| eye | gen_gwas_eye_no | 0.222911 | -0.13113 | 0.580324 | 1.249709 | 0.877101 | 1.786616 | 0.218795 | | 0.244383 | -0.14241 | 0.635587 | 1.276833 | 0.867262 | 1.8881308 | 0.217465 | | NA | NA | NA | NA |
| gi | gen_gwas_gi_no | 0.171583 | -0.07477 | 0.418337 | 1.187183 | 0.927956 | 1.519433 | 0.172369 | | 0.206053 | -0.06418 | 0.476928 | 1.228818 | 0.937835 | 1.611117 | 0.135289 | | NA | NA | NA | NA |
| hepatobiliary | gen_gwas_hepatobiliary_no | 0.363878 | -0.02841 | 0.762437 | 1.438899 | 0.97199 | 2.143494 | 0.070843 | | 0.318192 | -0.10007 | 0.743142 | 1.37464 | 0.904772 | 2.1025311 | 0.138343 | | NA | NA | NA | NA |
| immune | gen_gwas_immune_no | 0.015307 | -0.30534 | 0.335707 | 1.015425 | 0.73687 | 1.398929 | 0.925365 | | -0.11551 | -0.55926 | 0.328036 | 0.890915 | 0.571632 | 1.3882396 | 0.60927 | | NA | NA | NA | NA |
| infection | gen_gwas_infection_no | 0.244918 | -0.00694 | 0.498062 | 1.277517 | 0.993086 | 1.64553 | 0.057158 | | 0.257716 | -0.00893 | 0.525931 | 1.293971 | 0.991107 | 1.6920326 | 0.058772 | | NA | NA | NA | NA |
| metabolism | gen_gwas_metabolism_no | 0.116282 | -0.17323 | 0.405294 | 1.123313 | 0.840941 | 1.499744 | 0.430443 | | 0.216994 | -0.12709 | 0.561864 | 1.242337 | 0.880651 | 1.753939 | 0.216509 | | NA | NA | NA | NA |
| musculoskeletal | gen_gwas_musculoskeletal_no | 0.043324 | -0.21715 | 0.303843 | 1.044276 | 0.804809 | 1.355056 | 0.744309 | | 0.076667 | -0.22573 | 0.379333 | 1.079682 | 0.797935 | 1.4613092 | 0.619136 | | NA | NA | NA | NA |
| neoplasm | gen_gwas_neoplasm_no | -0.02746 | -0.45743 | 0.402286 | 0.972909 | 0.632908 | 1.495238 | 0.900173 | | -0.02746 | -0.45743 | 0.402286 | 0.972909 | 0.632908 | 1.4952382 | 0.900173 | | NA | NA | NA | NA |
| nervous | gen_gwas_nervous_no | 0.031054 | -0.2095 | 0.271275 | 1.031541 | 0.810988 | 1.311636 | 0.80004 | | -0.20306 | -0.49095 | 0.083172 | 0.816231 | 0.612044 | 1.0867288 | 0.165358 | | NA | NA | NA | NA |
| pregnancy | gen_gwas_pregnancy_no | 0.854052 | 0.095443 | 1.656801 | 2.349146 | 1.100146 | 5.242514 | 0.030914 | | 1.099616 | 0.271011 | 1.993582 | 3.003014 | 1.311289 | 7.3417838 | 0.011664 | | NA | NA | NA | NA |
| mental | gen_gwas_mental_no | -0.11378 | -0.4059 | 0.176855 | 0.892454 | 0.666376 | 1.193458 | 0.443734 | | -0.09244 | -0.42892 | 0.242449 | 0.911707 | 0.651214 | 1.2743662 | 0.589008 | | NA | NA | NA | NA |
| urologic | gen_gwas_urologic_no | 0.285575 | -0.0431 | 0.616815 | 1.330527 | 0.957816 | 1.853016 | 0.089501 | | 0.266717 | -0.09996 | 0.636713 | 1.305671 | 0.904874 | 1.890258 | 0.155294 | | NA | NA | NA | NA |
| repro | gen_gwas_repro_no | 0.182218 | -0.17715 | 0.543505 | 1.199876 | 0.837656 | 1.722032 | 0.321005 | | 0.204212 | -0.18628 | 0.596753 | 1.226558 | 0.830044 | 1.8162115 | 0.305911 | | NA | NA | NA | NA |
| respiratory | gen_gwas_respiratory_no | 0.28734 | 0.031085 | 0.544987 | 1.332877 | 1.031573 | 1.724587 | 0.02831 | | 0.350579 | 0.072679 | 0.63048 | 1.419889 | 1.075386 | 1.8785126 | 0.01369 | | NA | NA | NA | NA |
| skin | gen_gwas_skin_no | -0.04879 | -0.30734 | 0.209843 | 0.952379 | 0.735402 | 1.233484 | 0.711361 | | -0.03205 | -0.31406 | 0.250091 | 0.968463 | 0.730478 | 1.2841427 | 0.823679 | | NA | NA | NA | NA |
| vascular | gen_gwas_vascular_no | 0.06188 | -0.1845 | 0.307699 | 1.063835 | 0.831522 | 1.360292 | 0.621975 | | 0.009343 | -0.26536 | 0.283141 | 1.009386 | 0.766932 | 1.3272921 | 0.946734 | | NA | NA | NA | NA |

**Supplementary Table 3.** Coefficients (as beta, and transformed to odds ratios) for genetics variables in side effect models. Confidence intervals and p values are outputs from the *glm* function in R.

| side effect modeled | AUC.real.genetics | AUC.no.genetics | median.AUC.random.genetics | CI95.AUC.random.genetics | p |
|---|---|---|---|---|---|
| heart | 0.703 | 0.684 | 0.682 | 0.675-0.695 | < 0.001 |
| metabolism | 0.706 | 0.701 | 0.691 | 0.687-0.7 | < 0.001 |
| repro | 0.731 | 0.72 | 0.712 | 0.706-0.725 | 0.006 |
| endocrine | 0.704 | 0.678 | 0.671 | 0.658-0.695 | 0.007 |
| urologic | 0.739 | 0.732 | 0.723 | 0.716-0.736 | 0.007 |
| mental | 0.712 | 0.716 | 0.702 | 0.697-0.714 | 0.037 |

**Supplementary Table 4.** Results of cross validataion analysis of six off-indication side effect models where genetics was significant in any of the regression models. Distribution of permutation values are shown in **Figure 4** and are reflected in 95% CI values. The p values are derived from the frequency of permutation runs that exceed the AUC of the true model.

| **Adverse Event** | **# drugs** | **% drugs** | **UMLS ID** |
|---|---|---|---|
| headache | 1446 | 54.9 | C0018681 |
| nausea | 1209 | 45.9 | C0027497 |
| diarrhea | 959 | 36.4 | C0011991 |
| emesis | 708 | 26.9 | C0042963 |
| dizziness | 617 | 23.4 | C0012833,C0042571 |
| fatigue | 607 | 23.1 | C0015672 |
| pain | 606 | 23.0 | C0030193 |
| rhinopharyngitis | 531 | 20.2 | C0027441 |
| upper respiratory tract infection | 498 | 18.9 | C0041912 |
| constipation | 462 | 17.5 | C0009806 |
| vertigo | 440 | 16.7 | C0042571 |
| skin rash | 422 | 16.0 | C0015230 |
| abdominal pain | 410 | 15.6 | C0000737 |
| gastrointestinal disease | 388 | 14.7 | C0017178 |
| fever | 386 | 14.7 | C0015967 |
| back pain | 381 | 14.5 | C0004604 |
| pruritus | 381 | 14.5 | C0033774 |
| drowsiness | 368 | 14.0 | C2830004 |
| death | 353 | 13.4 | C1306577 |
| insomnia | 339 | 12.9 | C0917801 |
| cough | 332 | 12.6 | C0010200 |
| dyspepsia | 327 | 12.4 | C0013395 |
| arthralgia | 322 | 12.2 | C0003862 |
| infectious disease | 316 | 12.0 | C0009450 |
| urinary tract infection | 307 | 11.7 | C0042029 |
| hypertension | 304 | 11.5 | C0020538 |
| edema | 285 | 10.8 | C0013604 |

**Supplementary Table 5**. Common side effects removed from analysis.

| enteral | parenteral | topical | other |
|---|---|---|---|
| buccal | epidural | dental | instillation, bladder |
| nasograstric | infiltration | inhaled | instillation, intravesical |
| oral | intraarterial | intranasal | intrabone |
| oro-pharingeal | intraarterial (hepatic artery) | intranasal spray | intrabronchial |
| rectal | intraarticular | mouth rinse | intracameral |
| sublingual | intracoronary | ophthalmic | intracardiac |
| | intracutaneous | percutaneous | intracavernosal |
| | intradermal | topical | intracerebral |
| | intralymphatic | topical (buccal) | intracerebroventricular |
| | intramuscular | topical (ophthalmic) | intradiscal |
| | intramuscular (cremaster) | topical (otic) | intragastric |
| | intramuscular (deltoid) | | intra-locus coeruleus |
| | intramuscular (gluteal) | | intramammary |
| | intraspinal | | intramyocardial |
| | intrathecal | | intranodal |
| | intrathecal, lumbar | | intraocular |
| | intravenous | | intraotic |
| | intravenous (portal) | | intrapericardial |
| | parenteral | | intraperitoneal |
| | subcutaneous | | intrapleural |
| | subcutaneous (abdomen) | | intraprostatic |
| | subcutaneous (arm) | | intrastriatal |
| | subcutaneous (thigh) | | intratendinous |
| | transarterial | | intratracheal |
| | transdermal | | intratumor |
| | | | intratumoral |
| | | | intratympanic |
| | | | intrauterine |
| | | | intravaginal |
| | | | intraventricular |
| | | | intravitreous |
| | | | percutaneous hepatic perfusion |
| | | | peritumoral |
| | | | subconjunctiva |
| | | | subgingivally |
| | | | subretinal |
| | | | subtenon |
| | | | urethral |

**Supplementary Table 6.** Coding of delivery route.

| Organ System Abbreviation | MedDRA System Organ Class (SOC) Term | UMLS id | Comment |
|---|---|---|---|
| blood | Blood and lymphatic system disorders | C0851353 | |
| heart | Diseases, Heart | C0018799 | |
| congenital | Congenital, familial and genetic disorders | C0851352 | |
| ear | Ear and labyrinth disorders | C0851354 | |
| endocrine | System Diseases, Endocrine | C0014130 | |
| eye | Diseases, Eye | C0015397 | |
| gi | Diseases, Gastrointestinal | C0017178 | |
| general | General disorders and administration site conditions | C0851362 | mapped to other SOC terms |
| hepatobiliary | Hepatobiliary disease NOS | C0267792 | |
| immune | Diseases, Immune System | C0021053 | |
| infection | Diseases, Communicable | C0009450 | |
| injury | Injury, poisoning and procedural complications | C0947733 | mapped to other SOC terms |
| investigation | Investigation NOS | C1261322 | mapped to other SOC terms |
| metabolism | Metabolism and nutrition disorders | C0851358 | |
| musculoskeletal | Musculoskeletal and connective tissue disorders | C0263660 | |
| neoplasm | Neoplasm NOS | C0027651 | |
| nervous | Diseases, Nervous System | C0027765 | |
| pregnancy | Pregnancy, puerperium and perinatal conditions | C0851363 | |
| mental | Mental disorder | C0004936 | |
| urologic | Diseases, Urologic | C0042075 | |
| repro | Reproductive system and breast disorders | C0851366 | |
| respiratory | Respiratory, thoracic and mediastinal disorders | C0851355 | |
| skin | Skin and subcutaneous tissue disorders | C0178298 | |
| social | Social circumstances | C0851364 | exclude from study |
| procedures | Surgical and medical procedures | C1948041 | exclude from study |
| vascular | Diseases, Vascular | C0042373 | |

**Supplementary Table 7.** MedDRA System Organ Class (SOC) terms inclusion and remapping criteria.

| | | This study (human x 21 organ systems) | IQ Consortium (3 species x 12 organ systems) |
|---|---|---|---|
| **Translatability of positive findings** | | | |
| Number of species-phenotype combinations with: | LR+ ≤ 1 | 1 | 7 |
| | 1 < LR+ < 10 | 20 | 22 |
| | LR+ ≥ 10 | 0 | 4 |
| Sensitivity | | 30% | 48% |
| PPV | | 30% | 43% |
| **Translatability of negative findings** | | | |
| Number of species-phenotype combinations with: | iLR- ≤ 1 | 1 | 10 |
| | 1 < iLR- < 10 | 20 | 25 |
| | iLR- ≥ 10 | 0 | 0 |
| Specificity | | 80% | 84% |
| NPV | | 81% | 86% |

**Supplementary Table 8.** Comparison of summary statistics between results of this study and the results of the IQ Consortium (Monticello *et al.*, 2017). PPV, positive predictive value. NPV, negative predictive value. LR+, positive likelihood ratio. iLR-, inverse negative likelihood ratio.

| | | Model: glm (all data used) | | | | | | | | Model: glm (only off-indication side effects used) | | | | | | | | Model: glmnet (all data used) | | | Model:glmnet (only off-indication data used) | |
|---|---|---|---|---|---|---|---|---|---|---|---|---|---|---|---|---|---|---|---|---|---|---|
| Model (Side effect predicted) | Variable | beta | 95% CI | | OR | 95% CI | | p | | beta | 95% CI | | OR | 95% CI | | p | | beta | OR | | beta | OR |
| blood | gen_omim_blood_yes | 0.162092 | -0.3047 | 0.621131 | 1.175968 | 0.737347 | 1.861032 | 0.491958 | | 0.091146 | -0.42316 | 0.59299 | 1.095429 | 0.654972 | 1.80939 | 0.724693 | | NA | NA | | NA | NA |
| heart | gen_omim_heart_yes | 0.709114 | 0.270634 | 1.14718 | 2.03219 | 1.310796 | 3.1493 | 0.001505 | | 0.838638 | 0.359089 | 1.316276 | 2.313214 | 1.432024 | 3.729507 | 0.000586 | | 0.287803 | 1.333495 | | 0.325952305 | 1.385349293 |
| congenital | gen_omim_congenital_yes | 0.935395 | -0.26439 | 2.229239 | 2.548219 | 0.767676 | 9.292789 | 0.13694 | | 2.345718 | 0.124561 | 5.306846 | 10.44077 | 1.132651 | 201.7131 | 0.063 | | NA | NA | | NA | NA |
| ear | gen_omim_ear_yes | 0.184921 | -1.08308 | 1.283147 | 1.203124 | 0.338553 | 3.607978 | 0.755659 | | -0.01987 | -1.39101 | 1.135715 | 0.980325 | 0.248823 | 3.113398 | 0.974913 | | NA | NA | | NA | NA |
| endocrine | gen_omim_endocrine_yes | 0.952153 | 0.421856 | 1.482671 | 2.591283 | 1.524789 | 4.404696 | 0.000426 | | 1.196117 | 0.478165 | 1.907841 | 3.307249 | 1.613112 | 6.738521 | 0.000998 | | 0.217877 | 1.243434 | | 0.154896338 | 1.167536926 |
| eye | gen_omim_eye_yes | -0.06875 | -0.63011 | 0.477691 | 0.933563 | 0.532535 | 1.612347 | 0.807445 | | -0.15367 | -0.79461 | 0.464039 | 0.857556 | 0.451755 | 1.590485 | 0.631254 | | NA | NA | | NA | NA |
| gi | gen_omim_gi_yes | -0.29415 | -0.65022 | 0.057828 | 0.745167 | 0.521929 | 1.059533 | 0.10319 | | -0.10457 | -0.4915 | 0.27835 | 0.900714 | 0.611708 | 1.320948 | 0.594114 | | NA | NA | | NA | NA |
| hepatobiliary | gen_omim_hepatobiliary_yes | -0.293 | -1.1182 | 0.479707 | 0.746022 | 0.326867 | 1.615602 | 0.470619 | | -0.64547 | -1.68957 | 0.280241 | 0.524418 | 0.1846 | 1.323449 | 0.195131 | | NA | NA | | NA | NA |
| immune | gen_omim_immune_yes | 0.278185 | -0.14869 | 0.698569 | 1.320731 | 0.861839 | 2.010873 | 0.197499 | | 0.046765 | -0.68232 | 0.73583 | 1.047875 | 0.505443 | 2.087213 | 0.896641 | | NA | NA | | NA | NA |
| infection | gen_omim_infection_yes | 0.233047 | -0.26189 | 0.722432 | 1.262441 | 0.769594 | 2.059435 | 0.352636 | | 0.238231 | -0.29628 | 0.766419 | 1.269002 | 0.743579 | 2.152046 | 0.378714 | | NA | NA | | NA | NA |
| metabolism | gen_omim_metabolism_yes | 0.636062 | 0.298069 | 0.97566 | 1.889027 | 1.347254 | 2.652917 | 0.000231 | | 0.527928 | 0.08995 | 0.965871 | 1.695416 | 1.09412 | 2.627075 | 0.018008 | | 0.084695 | 1.088385 | | 0.011376563 | 1.011441523 |
| musculoskeletal | gen_omim_musculoskeletal_yes | 0.214049 | -0.09963 | 0.527754 | 1.238684 | 0.905175 | 1.69512 | 0.180807 | | 0.2859 | -0.08112 | 0.653439 | 1.33096 | 0.922084 | 1.92214 | 0.126746 | | NA | NA | | 0.039245178 | 1.040025443 |
| neoplasm | gen_omim_neoplasm_yes | 0.533812 | -0.13093 | 1.188392 | 1.705421 | 0.87728 | 3.281801 | 0.111976 | | 0.533812 | -0.13093 | 1.188392 | 1.705421 | 0.87728 | 3.281801 | 0.111976 | | NA | NA | | NA | NA |
| nervous | gen_omim_nervous_yes | -0.01654 | -0.3053 | 0.271444 | 0.983596 | 0.736905 | 1.311857 | 0.910448 | | 0.267309 | -0.09063 | 0.625795 | 1.306444 | 0.913354 | 1.869732 | 0.14326 | | NA | NA | | NA | NA |
| pregnancy | gen_omim_pregnancy_yes | 0.311179 | -1.01433 | 1.568157 | 1.365033 | 0.362644 | 4.797795 | 0.632798 | | 0.559077 | -0.86173 | 1.924256 | 1.749058 | 0.42243 | 6.850052 | 0.426027 | | NA | NA | | NA | NA |
| mental | gen_omim_mental_yes | 0.353576 | -0.01715 | 0.723029 | 1.424151 | 0.982991 | 2.060665 | 0.060926 | | 0.371056 | -0.08904 | 0.82766 | 1.449264 | 0.914806 | 2.287959 | 0.112109 | | 0.163284 | 1.177371 | | NA | NA |
| urologic | gen_omim_urologic_yes | 0.591618 | 0.115107 | 1.06582 | 1.806909 | 1.121993 | 2.903218 | 0.014587 | | 0.723119 | 0.181844 | 1.260413 | 2.060851 | 1.199427 | 3.526876 | 0.00846 | | 0.11313 | 1.119777 | | 0.296989895 | 1.3458017 |
| repro | gen_omim_repro_yes | 0.993491 | 0.428062 | 1.551227 | 2.700646 | 1.534282 | 4.717257 | 0.000513 | | 0.913327 | 0.27293 | 1.538904 | 2.492601 | 1.313808 | 4.65948 | 0.004574 | | 0.575129 | 1.777359 | | 0.328021306 | 1.38821855 |
| respiratory | gen_omim_respiratory_yes | 0.142477 | -0.21035 | 0.493275 | 1.153126 | 0.8103 | 1.637671 | 0.427019 | | 0.027181 | -0.36721 | 0.41789 | 1.027553 | 0.692664 | 1.518753 | 0.891956 | | NA | NA | | NA | NA |
| skin | gen_omim_skin_yes | -0.07187 | -0.45144 | 0.303625 | 0.930655 | 0.636711 | 1.354761 | 0.708848 | | -0.08137 | -0.49811 | 0.329332 | 0.921856 | 0.607676 | 1.390039 | 0.699591 | | NA | NA | | NA | NA |
| vascular | gen_omim_vascular_yes | 0.196397 | -0.13257 | 0.524479 | 1.21701 | 0.875839 | 1.689579 | 0.241024 | | 0.086637 | -0.3011 | 0.47102 | 1.090501 | 0.740006 | 1.601626 | 0.65975 | | 0.016293 | 1.016426 | | NA | NA |
| blood | gen_omim_blood_no | -0.06826 | -0.41805 | 0.281478 | 0.934015 | 0.658332 | 1.325087 | 0.701802 | | -0.07765 | -0.44624 | 0.290561 | 0.925292 | 0.640033 | 1.337178 | 0.679215 | | NA | NA | | NA | NA |
| heart | gen_omim_heart_no | 0.18758 | -0.08777 | 0.464246 | 1.206327 | 0.91597 | 1.590814 | 0.182585 | | 0.217087 | -0.08664 | 0.522831 | 1.242452 | 0.917011 | 1.686795 | 0.162311 | | NA | NA | | NA | NA |
| congenital | gen_omim_congenital_no | 0.556453 | -0.75215 | 1.875506 | 1.744474 | 0.471353 | 6.524119 | 0.400224 | | -0.429 | -3.69902 | 2.711292 | 0.651157 | 0.024748 | 15.04871 | 0.786016 | | NA | NA | | NA | NA |
| ear | gen_omim_ear_no | 0.283705 | -0.36565 | 0.951975 | 1.328042 | 0.693746 | 2.59082 | 0.396474 | | 0.290216 | -0.3766 | 0.977593 | 1.336716 | 0.686187 | 2.658051 | 0.398499 | | NA | NA | | NA | NA |
| endocrine | gen_omim_endocrine_no | 0.435031 | 0.012475 | 0.863699 | 1.545012 | 1.012554 | 2.371919 | 0.044802 | | 0.488916 | -0.04701 | 1.03844 | 1.630547 | 0.954077 | 2.824806 | 0.076558 | | NA | NA | | NA | NA |
| eye | gen_omim_eye_no | 0.047614 | -0.35236 | 0.449448 | 1.048766 | 0.703028 | 1.567447 | 0.815684 | | 0.031798 | -0.42396 | 0.489774 | 1.032309 | 0.65445 | 1.631947 | 0.891298 | | NA | NA | | NA | NA |
| gi | gen_omim_gi_no | -0.19492 | -0.47221 | 0.081635 | 0.822901 | 0.623622 | 1.08506 | 0.167483 | | -0.08399 | -0.38774 | 0.219655 | 0.919442 | 0.678591 | 1.245646 | 0.587535 | | NA | NA | | NA | NA |
| hepatobiliary | gen_omim_hepatobiliary_no | -0.15745 | -0.59371 | 0.28023 | 0.854317 | 0.552273 | 1.323434 | 0.479237 | | -0.01814 | -0.48015 | 0.448807 | 0.982025 | 0.618691 | 1.566442 | 0.938856 | | NA | NA | | NA | NA |
| immune | gen_omim_immune_no | 0.179531 | -0.16292 | 0.523773 | 1.196655 | 0.849661 | 1.688386 | 0.305004 | | 0.034616 | -0.44613 | 0.522257 | 1.035222 | 0.6401 | 1.685828 | 0.888343 | | NA | NA | | NA | NA |
| infection | gen_omim_infection_no | 0.035065 | -0.24121 | 0.312199 | 1.035687 | 0.785677 | 1.366426 | 0.803738 | | -0.02538 | -0.31735 | 0.267362 | 0.974942 | 0.728073 | 1.306513 | 0.864824 | | NA | NA | | NA | NA |
| metabolism | gen_omim_metabolism_no | 0.197369 | -0.14211 | 0.536591 | 1.218193 | 0.867528 | 1.710167 | 0.253936 | | 0.22929 | -0.1657 | 0.624887 | 1.257707 | 0.847297 | 1.868034 | 0.255031 | | NA | NA | | NA | NA |
| musculoskeletal | gen_omim_musculoskeletal_no | -0.08626 | -0.39685 | 0.222583 | 0.917359 | 0.672435 | 1.2493 | 0.584903 | | -0.08239 | -0.44194 | 0.274764 | 0.92091 | 0.642789 | 1.31622 | 0.651931 | | NA | NA | | NA | NA |
| neoplasm | gen_omim_neoplasm_no | -0.20252 | -0.69404 | 0.287333 | 0.816667 | 0.499554 | 1.332867 | 0.417514 | | -0.20252 | -0.69404 | 0.287333 | 0.816667 | 0.499554 | 1.332867 | 0.417514 | | NA | NA | | NA | NA |
| nervous | gen_omim_nervous_no | 0.123526 | -0.17412 | 0.420969 | 1.131479 | 0.8402 | 1.523437 | 0.415581 | | 0.209155 | -0.13489 | 0.553539 | 1.232635 | 0.873812 | 1.739397 | 0.233317 | | NA | NA | | NA | NA |
| pregnancy | gen_omim_pregnancy_no | -0.21143 | -1.1365 | 0.706452 | 0.809426 | 0.32094 | 2.026786 | 0.650078 | | -0.24671 | -1.24838 | 0.746712 | 0.781365 | 0.286968 | 2.11005 | 0.624435 | | NA | NA | | NA | NA |
| mental | gen_omim_mental_no | 0.076743 | -0.27227 | 0.426049 | 1.079764 | 0.761648 | 1.531196 | 0.666353 | | 0.2366 | -0.15859 | 0.634078 | 1.266934 | 0.85335 | 1.885283 | 0.241378 | | NA | NA | | NA | NA |
| urologic | gen_omim_urologic_no | -0.06371 | -0.45245 | 0.325373 | 0.938276 | 0.636068 | 1.384547 | 0.747837 | | -0.03926 | -0.47496 | 0.39765 | 0.961501 | 0.621908 | 1.488323 | 0.859769 | | NA | NA | | NA | NA |
| repro | gen_omim_repro_no | -0.18886 | -0.62197 | 0.243867 | 0.827903 | 0.536884 | 1.276174 | 0.391827 | | -0.15285 | -0.62701 | 0.32164 | 0.858262 | 0.534189 | 1.379388 | 0.526901 | | NA | NA | | NA | NA |
| respiratory | gen_omim_respiratory_no | 0.017435 | -0.28374 | 0.318731 | 1.017588 | 0.752966 | 1.375381 | 0.909625 | | -0.0855 | -0.41672 | 0.245148 | 0.918052 | 0.659204 | 1.277811 | 0.612323 | | NA | NA | | NA | NA |
| skin | gen_omim_skin_no | -0.24749 | -0.54618 | 0.05007 | 0.78076 | 0.579156 | 1.051345 | 0.103498 | | -0.30631 | -0.63671 | 0.022203 | 0.73616 | 0.529032 | 1.022451 | 0.068186 | | NA | NA | | NA | NA |
| vascular | gen_omim_vascular_no | 0.017479 | -0.25792 | 0.292845 | 1.017632 | 0.772658 | 1.340235 | 0.900945 | | -0.05025 | -0.3606 | 0.259874 | 0.950993 | 0.697255 | 1.296766 | 0.750731 | | NA | NA | | NA | NA |
| blood | gen_gwas_blood_yes | 0.354919 | -0.14976 | 0.850343 | 1.426065 | 0.860912 | 2.34045 | 0.163569 | | 0.588198 | 0.038603 | 1.127301 | 1.80074 | 1.039358 | 3.087313 | 0.033845 | | NA | NA | | NA | NA |
| heart | gen_gwas_heart_yes | 0.713666 | 0.258691 | 1.16798 | 2.041461 | 1.295233 | 3.215489 | 0.002073 | | 0.798489 | 0.300843 | 1.293995 | 2.22218 | 1.350997 | 3.647328 | 0.001604 | | 0.326082 | 1.38553 | | 0.411220872 | 1.50865854 |
| congenital | gen_gwas_congenital_yes | 1.375561 | -0.3206 | 2.978908 | 3.957297 | 0.725716 | 19.66634 | 0.096448 | | -18.9322 | NA | 132.4311 | 6.00E-09 | NA | 3.27E+57 | 0.994258 | | NA | NA | | NA | NA |
| ear | gen_gwas_ear_yes | -14.9689 | NA | 93.37754 | 3.16E-07 | NA | 3.58E+40 | 0.977696 | | -14.911 | NA | 93.39451 | 3.34E-07 | NA | 3.64E+40 | 0.977782 | | NA | NA | | NA | NA |
| endocrine | gen_gwas_endocrine_yes | 0.325005 | -0.35164 | 0.996775 | 1.384038 | 0.703535 | 2.709529 | 0.344111 | | -0.2471 | -1.30438 | 0.754428 | 0.781066 | 0.271339 | 2.126396 | 0.6366 | | NA | NA | | NA | NA |
| eye | gen_gwas_eye_yes | 0.63102 | -0.11954 | 1.352384 | 1.879528 | 0.887331 | 3.866634 | 0.092035 | | 0.276686 | -0.67362 | 1.148453 | 1.318752 | 0.509861 | 3.15331 | 0.549397 | | NA | NA | | NA | NA |
| gi | gen_gwas_gi_yes | -0.02087 | -0.52676 | 0.471569 | 0.979346 | 0.590514 | 1.602506 | 0.934585 | | 0.242315 | -0.306 | 0.776122 | 1.274196 | 0.736384 | 2.173028 | 0.379176 | | NA | NA | | NA | NA |
| hepatobiliary | gen_gwas_hepatobiliary_yes | 0.436959 | -0.69645 | 1.448954 | 1.547993 | 0.498353 | 4.258659 | 0.42 | | 0.582093 | -0.59097 | 1.630988 | 1.78978 | 0.553788 | 5.108921 | 0.299306 | | NA | NA | | NA | NA |
| immune | gen_gwas_immune_yes | 0.117141 | -0.30361 | 0.530757 | 1.124277 | 0.738146 | 1.700219 | 0.581612 | | 0.197599 | -0.43835 | 0.810552 | 1.218474 | 0.645099 | 2.24915 | 0.53365 | | NA | NA | | NA | NA |
| infection | gen_gwas_infection_yes | -0.50691 | -1.31933 | 0.24122 | 0.602354 | 0.267314 | 1.2728 | 0.200583 | | -0.57396 | -1.55086 | 0.307662 | 0.563291 | 0.212065 | 1.360242 | 0.223055 | | -0.06381 | 0.938186 | | NA | NA |
| metabolism | gen_gwas_metabolism_yes | 0.387763 | 0.008205 | 0.766002 | 1.47368 | 1.008239 | 2.15115 | 0.04471 | | 0.673393 | 0.160949 | 1.182539 | 1.960879 | 1.174625 | 3.262648 | 0.009668 | | NA | NA | | NA | NA |
| musculoskeletal | gen_gwas_musculoskeletal_yes | -0.30399 | -0.77676 | 0.155086 | 0.737871 | 0.459894 | 1.167758 | 0.200355 | | -0.19664 | -0.73299 | 0.321918 | 0.821485 | 0.480471 | 1.379772 | 0.464127 | | NA | NA | | NA | NA |
| neoplasm | gen_gwas_neoplasm_yes | 0.225651 | -0.52521 | 0.944184 | 1.253139 | 0.591428 | 2.570714 | 0.545972 | | 0.225651 | -0.52521 | 0.944184 | 1.253139 | 0.591428 | 2.570714 | 0.545972 | | NA | NA | | NA | NA |
| nervous | gen_gwas_nervous_yes | -0.01921 | -0.40654 | 0.36527 | 0.980977 | 0.665953 | 1.440902 | 0.92223 | | -0.15743 | -0.63971 | 0.317321 | 0.854335 | 0.527447 | 1.373444 | 0.518533 | | NA | NA | | NA | NA |
| pregnancy | gen_gwas_pregnancy_yes | -13.1002 | -354.394 | 30.43052 | 2.04E-06 | 1.23E-154 | 1.64E+13 | 0.98984 | | -12.5246 | -346.384 | 30.97088 | 3.64E-06 | 3.69E-151 | 2.82E+13 | 0.990157 | | NA | NA | | NA | NA |
| mental | gen_gwas_mental_yes | 0.360976 | -0.09129 | 0.80976 | 1.43473 | 0.912752 | 2.247368 | 0.116007 | | 0.325475 | -0.23716 | 0.878354 | 1.384688 | 0.788866 | 2.406935 | 0.251995 | | 0.269078 | 1.308757 | | 0.082018097 | 1.085475454 |
| urologic | gen_gwas_urologic_yes | 0.391469 | -1.19961 | 1.682431 | 1.479152 | 0.301311 | 5.378615 | 0.587261 | | 0.866324 | -0.6407 | 2.125675 | 2.378152 | 0.526925 | 8.378547 | 0.211185 | | NA | NA | | NA | NA |
| repro | gen_gwas_repro_yes | 0.856832 | 0.134536 | 1.571122 | 2.355686 | 1.144006 | 4.812043 | 0.019206 | | 0.499773 | -0.38899 | 1.349439 | 1.648347 | 0.677744 | 3.855261 | 0.257918 | | 0.150329 | 1.162216 | | NA | NA |
| respiratory | gen_gwas_respiratory_yes | 0.00276 | -0.76588 | 0.732457 | 1.002764 | 0.464923 | 2.080184 | 0.99422 | | -0.08004 | -1.03061 | 0.780216 | 0.923077 | 0.356789 | 2.181943 | 0.861441 | | NA | NA | | NA | NA |
| skin | gen_gwas_skin_yes | 0.506035 | -0.19968 | 1.198747 | 1.658701 | 0.818992 | 3.31596 | 0.154916 | | 0.468862 | -0.34964 | 1.251718 | 1.598175 | 0.70494 | 3.496343 | 0.248889 | | NA | NA | | NA | NA |
| vascular | gen_gwas_vascular_yes | 0.230687 | -0.15872 | 0.61794 | 1.259465 | 0.853232 | 1.855103 | 0.243914 | | 0.252312 | -0.2203 | 0.716826 | 1.286998 | 0.802281 | 2.047922 | 0.290502 | | NA | NA | | NA | NA |
| blood | gen_gwas_blood_no | 0.518247 | 0.172945 | 0.867042 | 1.679082 | 1.188801 | 2.379862 | 0.003396 | | 0.603241 | 0.23046 | 0.981304 | 1.828034 | 1.259179 | 2.667932 | 0.001619 | | NA | NA | | NA | NA |
| heart | gen_gwas_heart_no | 0.312906 | 0.036227 | 0.591103 | 1.367393 | 1.036892 | 1.805979 | 0.026973 | | 0.422683 | 0.119162 | 0.728682 | 1.52605 | 1.126552 | 2.072348 | 0.006519 | | NA | NA | | NA | NA |
| congenital | gen_gwas_congenital_no | 0.641124 | -0.40781 | 1.745746 | 1.898613 | 0.665106 | 5.730172 | 0.239304 | | -1.13879 | -3.44451 | 1.009898 | 0.320207 | 0.03192 | 2.74532 | 0.305653 | | NA | NA | | NA | NA |
| ear | gen_gwas_ear_no | 0.022093 | -0.61934 | 0.670513 | 1.022339 | 0.538301 | 1.955239 | 0.946291 | | 0.086066 | -0.57478 | 0.7556 | 1.089878 | 0.562827 | 2.128888 | 0.799086 | | NA | NA | | NA | NA |
| endocrine | gen_gwas_endocrine_no | 0.180966 | -0.20947 | 0.57452 | 1.198375 | 0.81101 | 1.776279 | 0.364931 | | 0.045755 | -0.44905 | 0.542871 | 1.046818 | 0.638235 | 1.72094 | 0.856194 | | NA | NA | | NA | NA |
| eye | gen_gwas_eye_no | 0.265352 | -0.13366 | 0.669477 | 1.30389 | 0.874886 | 1.953216 | 0.194648 | | 0.308827 | -0.12797 | 0.752914 | 1.361827 | 0.879882 | 2.123178 | 0.168599 | | NA | NA | | NA | NA |
| gi | gen_gwas_gi_no | 0.214063 | -0.05619 | 0.48524 | 1.2387 | 0.945355 | 1.624564 | 0.120985 | | 0.23841 | -0.06425 | 0.542139 | 1.26923 | 0.937772 | 1.719681 | 0.123015 | | NA | NA | | NA | NA |
| hepatobiliary | gen_gwas_hepatobiliary_no | 0.692472 | 0.240887 | 1.157495 | 1.998649 | 1.272377 | 3.181953 | 0.003011 | | 0.629181 | 0.149012 | 1.123258 | 1.876073 | 1.160687 | 3.074856 | 0.01119 | | NA | NA | | NA | NA |
| immune | gen_gwas_immune_no | 0.056089 | -0.28731 | 0.399596 | 1.057691 | 0.750275 | 1.491222 | 0.748695 | | -0.00784 | -0.48522 | 0.471166 | 0.992194 | 0.615564 | 1.601861 | 0.97433 | | NA | NA | | NA | NA |
| infection | gen_gwas_infection_no | 0.203814 | -0.06352 | 0.47248 | 1.22607 | 0.938457 | 1.603967 | 0.135859 | | 0.233104 | -0.04916 | 0.51715 | 1.262513 | 0.95203 | 1.677241 | 0.106409 | | NA | NA | | NA | NA |
| metabolism | gen_gwas_metabolism_no | 0.217149 | -0.09242 | 0.526995 | 1.24253 | 0.911726 | 1.693835 | 0.169072 | | 0.266234 | -0.10984 | 0.643297 | 1.305041 | 0.89598 | 1.902744 | 0.165363 | | NA | NA | | NA | NA |
| musculoskeletal | gen_gwas_musculoskeletal_no | 0.075156 | -0.20292 | 0.353634 | 1.078053 | 0.816343 | 1.424233 | 0.596338 | | 0.121616 | -0.20625 | 0.450479 | 1.12932 | 0.813633 | 1.569064 | 0.467527 | | NA | NA | | NA | NA |
| neoplasm | gen_gwas_neoplasm_no | 0.164188 | -0.32534 | 0.658913 | 1.178436 | 0.722284 | 1.93269 | 0.512396 | | 0.164188 | -0.32534 | 0.658913 | 1.178436 | 0.722284 | 1.93269 | 0.512396 | | NA | NA | | NA | NA |
| nervous | gen_gwas_nervous_no | 0.009146 | -0.25066 | 0.268803 | 1.009188 | 0.778286 | 1.308398 | 0.944949 | | -0.2104 | -0.52186 | 0.099933 | 0.810261 | 0.593414 | 1.105097 | 0.184383 | | NA | NA | | NA | NA |
| pregnancy | gen_gwas_pregnancy_no | 1.218645 | 0.319745 | 2.199971 | 3.382603 | 1.376777 | 9.024749 | 0.010458 | | 1.448162 | 0.464396 | 2.540115 | 4.255284 | 1.591053 | 12.68113 | 0.005842 | | NA | NA | | NA | NA |
| mental | gen_gwas_mental_no | -0.0304 | -0.34907 | 0.287874 | 0.97006 | 0.705347 | 1.333589 | 0.851482 | | 0.041489 | -0.3317 | 0.415662 | 1.042361 | 0.7177 | 1.515373 | 0.827532 | | NA | NA | | NA | NA |
| urologic | gen_gwas_urologic_no | 0.369457 | 0.006558 | 0.737157 | 1.446949 | 1.00658 | 2.089984 | 0.047187 | | 0.41922 | 0.008397 | 0.836829 | 1.520775 | 1.008432 | 2.309034 | 0.046962 | | NA | NA | | NA | NA |
| repro | gen_gwas_repro_no | 0.279904 | -0.12372 | 0.687945 | 1.323003 | 0.883624 | 1.989622 | 0.175842 | | 0.32728 | -0.11682 | 0.778491 | 1.38719 | 0.889744 | 2.178183 | 0.151128 | | NA | NA | | NA | NA |
| respiratory | gen_gwas_respiratory_no | 0.309267 | 0.03593 | 0.584401 | 1.362426 | 1.036583 | 1.793917 | 0.026992 | | 0.394382 | 0.092782 | 0.698712 | 1.483467 | 1.097222 | 2.011161 | 0.010675 | | NA | NA | | NA | NA |
| skin | gen_gwas_skin_no | 0.000245 | -0.28265 | 0.283763 | 1.000245 | 0.753786 | 1.328118 | 0.998648 | | 0.010519 | -0.29773 | 0.319692 | 1.010575 | 0.742501 | 1.376703 | 0.946711 | | NA | NA | | NA | NA |
| vascular | gen_gwas_vascular_no | 0.087091 | -0.17821 | 0.35244 | 1.090996 | 0.836766 | 1.422534 | 0.519773 | | 0.006054 | -0.29104 | 0.302836 | 1.006073 | 0.747484 | 1.353693 | 0.968101 | | NA | NA | | NA | NA |

**Supplementary Table 9.** Coefficients (as beta, and transformed to odds ratios) for genetics variables in side effect models with tissue-specific target gene expression included as quantitative variables. Confidence intervals and p values are outputs from the *glm* function in R.
