# Supplementary material for: Phenotypes associated with genes encoding drug targets are predictive of clinical trial side effects
Source: Nat Commun. 2019 Apr 5;10:1579. doi: 10.1038/s41467-019-09407-3 (PMC6450952; doi:10.1038/s41467-019-09407-3)
Supplement: Supplementary file 4 — Description of Additional Supplementary Files [file 41467_2019_9407_MOESM4_ESM.pdf]

Description of Additional Supplementary Information files

Supplementary Data 1.
Entire data set across 1,819 drugs used for enrichment and regression analysis.

Supplementary Data 2.
Validation dataset using side effects derived from OFFSIDES.

Supplementary Data 3.
Placebo-controlled dataset using side effects derived from AACT. Only side effects enriched in the drug-treatment arm are included.

Supplementary Data 4.
Placebo dataset using side effects derived from AACT. All side effects seen in placebo arms are included.

Supplementary Data 5.
Drug-arm dataset using side effects derived from AACT. All side effects seen in drug-treatment arms are included.
